# Supplementary material for: Identification and in-silico characterization of taxadien-5α-ol-O-acetyltransferase (TDAT) gene in Corylus avellana L
Source: PLoS One. 2021 Aug 27;16(8):e0256704. doi: 10.1371/journal.pone.0256704 (PMC8396717; doi:10.1371/journal.pone.0256704)
Supplement: S1 Fig — The arrows define the primers and the green rectangles illustrate nested-PCR products for each primer. (DOCX) [file pone.0256704.s001.docx]

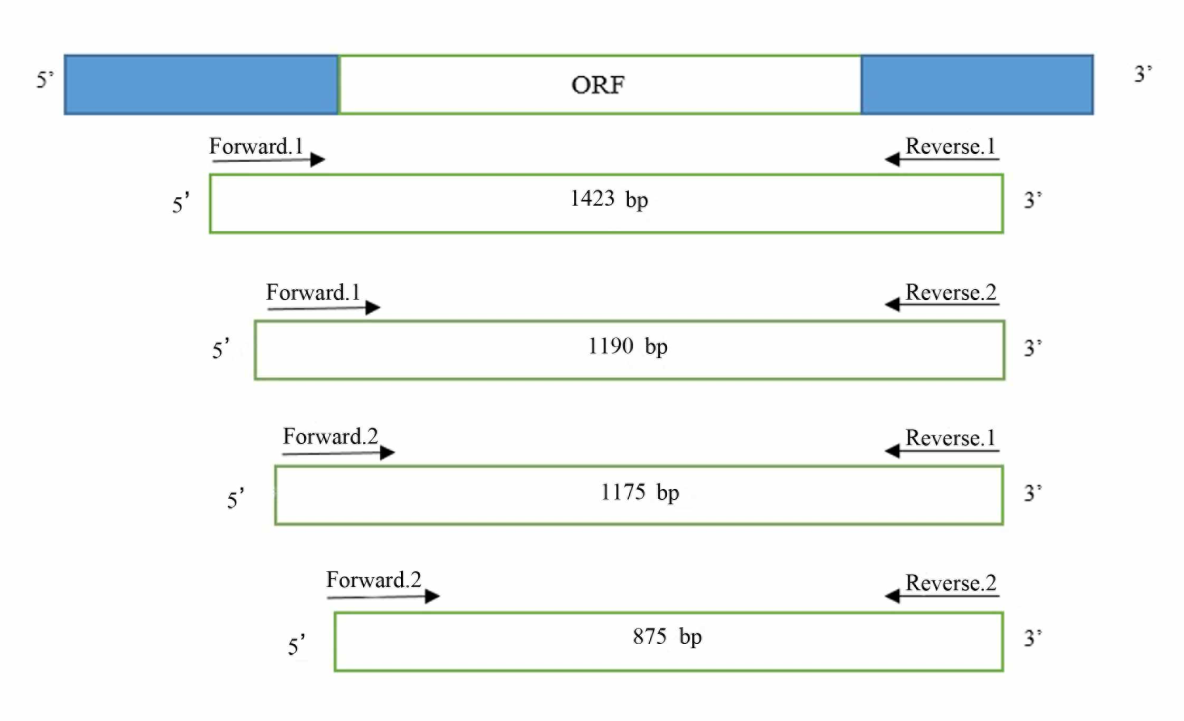


**S1 Fig. The position of full-length cDNA and nested-PCR primers containing *TDAT* gene.**

The arrows define the primers and the green rectangles illustrate nested-PCR products for each primer.
