# Supplementary material for: Identification and in-silico characterization of taxadien-5α-ol-O-acetyltransferase (TDAT) gene in Corylus avellana L
Source: PLoS One. 2021 Aug 27;16(8):e0256704. doi: 10.1371/journal.pone.0256704 (PMC8396717; doi:10.1371/journal.pone.0256704)
Supplement: S2 Fig — Evaluation of RT-PCR product of the different concentrations of MeJA induced cells and PCR purification. L: (ladder 100 bp), 1: Negative control (water), 2: RT-PCR products without any treatment (control), 3: RT-PCR product with 50 μM of MeJA, 4: RT-PCR product with 100 μM of MeJA, 5: RT-PCR product with 150 μM MeJA, 6: Negative control, 7: PCR product after PCR-purification in 150 μM of MeJA. The grouping of gels which have been cropped from different gels was identified with vertical white lines. (DOCX) [file pone.0256704.s002.docx]

**
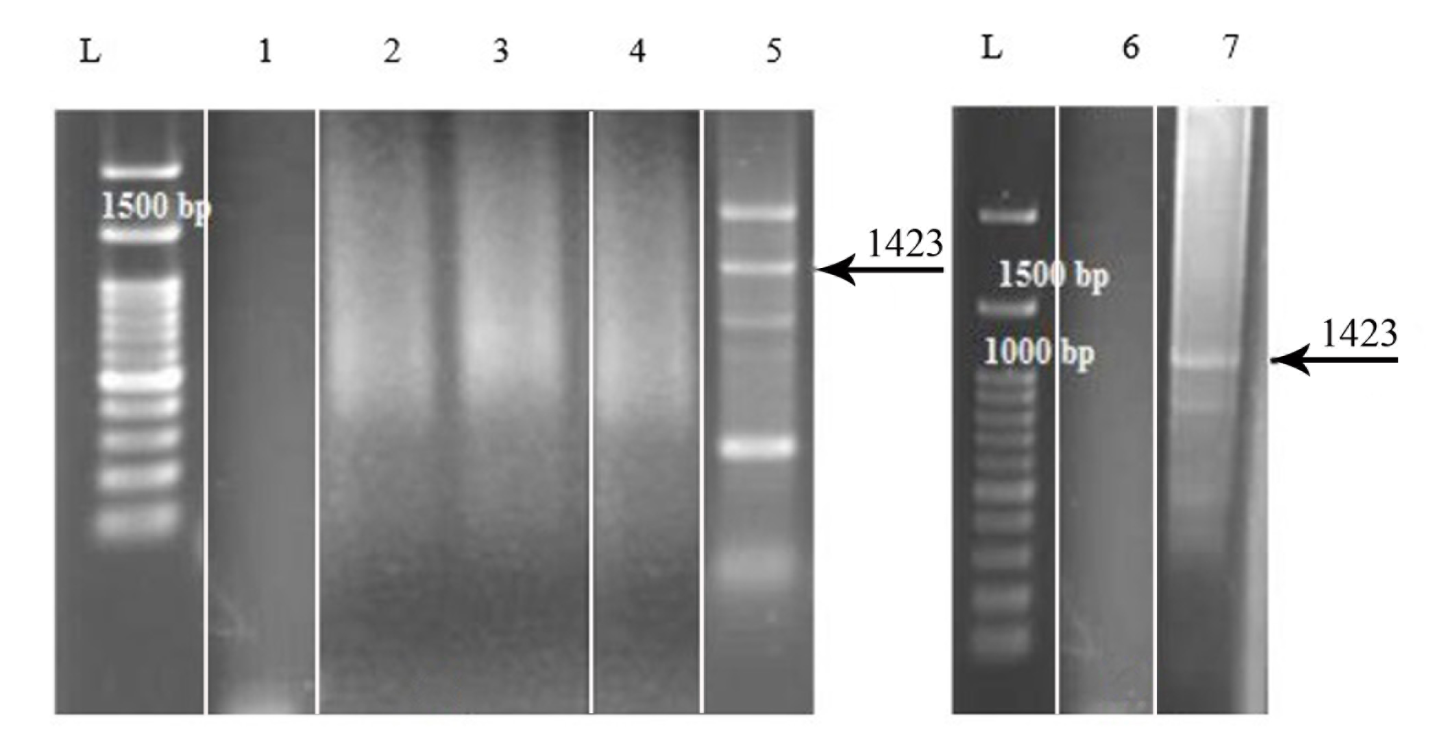
**

**S2 Fig.** **Agarose (1%) gel electrophoresis.**

Evaluation of RT-PCR product of the different concentrations of MeJA induced cells and PCR purification. L: (ladder 100 bp), 1: Negative control (water), 2: RT-PCR products without any treatment (control), 3: RT-PCR product with 50 µM of MeJA, 4: RT-PCR product with 100 µM of MeJA, 5: RT-PCR product with 150 µM MeJA, 6: Negative control, 7: PCR product after PCR-purification in 150 µM of MeJA. The grouping of gels which have been cropped from different gels was identified with vertical white lines.
