# Supplementary material for: Identification and in-silico characterization of taxadien-5α-ol-O-acetyltransferase (TDAT) gene in Corylus avellana L
Source: PLoS One. 2021 Aug 27;16(8):e0256704. doi: 10.1371/journal.pone.0256704 (PMC8396717; doi:10.1371/journal.pone.0256704)
Supplement: S3 Fig — Forward 1+ Reverse 2 (Fr1+Rr2), 3. Forward 2 + Reverse 1 (Fr2+Rr1), 4. Forward 2 + Reverse 2 (Fr2+Rr2), 5. Positive control (GAPDH primers), 6. Negative control (water). (DOCX) [file pone.0256704.s003.docx]

**
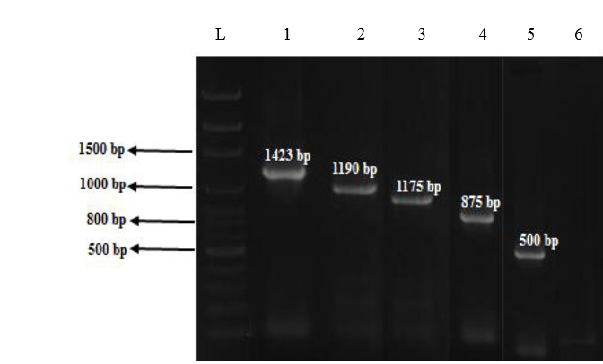
**

**S3 Fig. Agarose (1%) gel electrophoresis of nested-PCR analysis of *C. avellana*.**

Ladder (L) 100bp,1: Forward 1 + Reverse 1 (Fr_1_+Rr_1_), 2. Forward 1+ Reverse 2 (Fr_1_+Rr_2_), 3. Forward 2 + Reverse 1 (Fr_2_+Rr_1_), 4. Forward 2 + Reverse 2 (Fr_2_+Rr_2_), 5. Positive control (GAPDH primers), 6. Negative control (water).
