# Supplementary material for: Identification and in-silico characterization of taxadien-5α-ol-O-acetyltransferase (TDAT) gene in Corylus avellana L
Source: PLoS One. 2021 Aug 27;16(8):e0256704. doi: 10.1371/journal.pone.0256704 (PMC8396717; doi:10.1371/journal.pone.0256704)
Supplement: S4 Fig — (DOCX) [file pone.0256704.s004.docx]

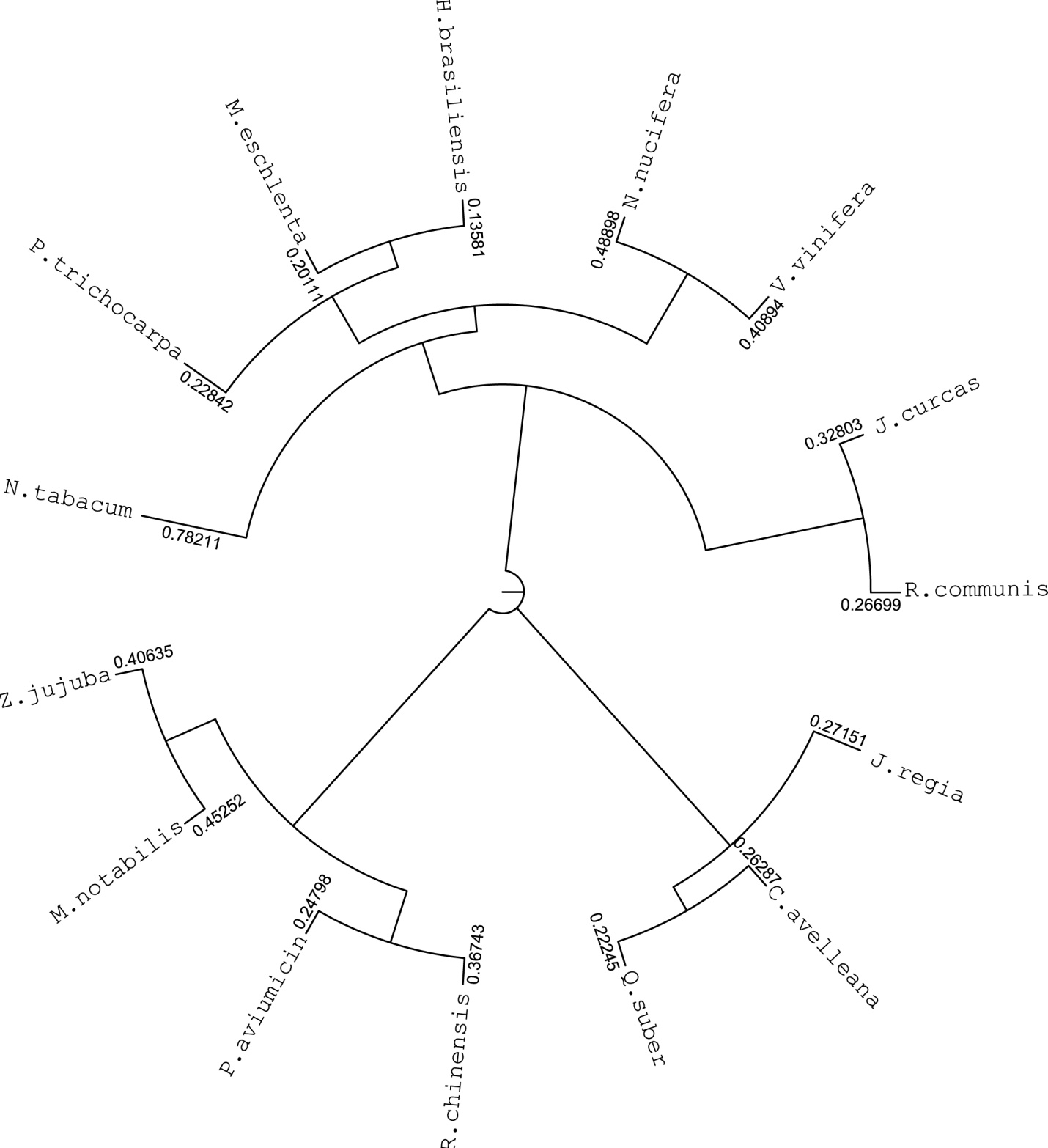


**S4 Fig. Evolutionary relationships of *C. avellana* L. *TDAT* gene with 15 geneses, based on the branch-site model, by using the EasyCodeML software (PAML package).**
