# Supplementary material for: Identification and in-silico characterization of taxadien-5α-ol-O-acetyltransferase (TDAT) gene in Corylus avellana L
Source: PLoS One. 2021 Aug 27;16(8):e0256704. doi: 10.1371/journal.pone.0256704 (PMC8396717; doi:10.1371/journal.pone.0256704)
Supplement: S5 Fig — All indicated parts with red diamonds are PBS. The regions are 1–4, 53–56, 116–120, 212–213, 397–397, and 408–410. (DOCX) [file pone.0256704.s005.docx]

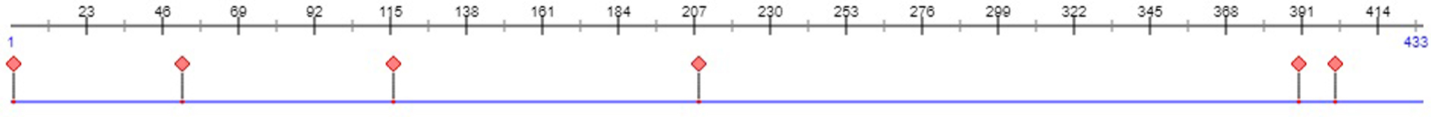


**S5 Fig.** **The protein binding sites (PBS) showed by PREDICT NLS. All indicated parts with red diamonds are PBS.**

The regions are 1 - 4, 53 - 56, 116 - 120, 212 - 213, 397 - 397, and 408 - 410.
