# Supplementary figures and images for: Identification and in-silico characterization of taxadien-5α-ol-O-acetyltransferase (TDAT) gene in Corylus avellana L
Source: PLoS One. 2021 Aug 27;16(8):e0256704. doi: 10.1371/journal.pone.0256704 (PMC8396717; doi:10.1371/journal.pone.0256704)

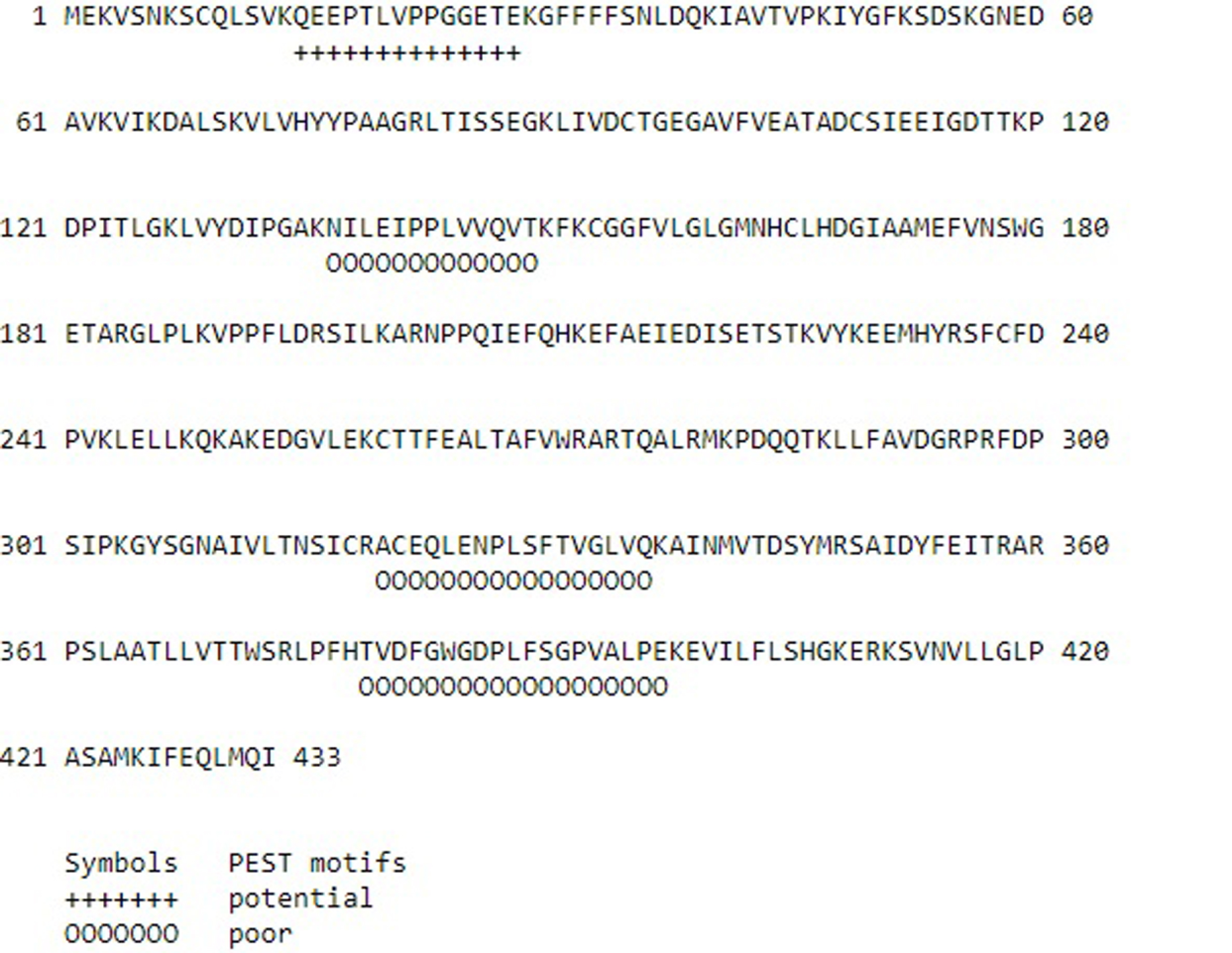


**S6 Fig.** **The position of the PEST motif was detected with Epestifind software.**

Supplement: S6 Fig — (DOCX) [file pone.0256704.s006.docx]
