# Supplementary material for: Identification and in-silico characterization of taxadien-5α-ol-O-acetyltransferase (TDAT) gene in Corylus avellana L
Source: PLoS One. 2021 Aug 27;16(8):e0256704. doi: 10.1371/journal.pone.0256704 (PMC8396717; doi:10.1371/journal.pone.0256704)
Supplement: S7 Fig — (A) 4G0B template (B) MO323. In the picture, [A, B, L] show a very favored region for amino acids. [a, b, l, p] show the allowed zone, and [~a, ~b, ~l, ~p] show the permitted regions with ignorance. (DOCX) [file pone.0256704.s007.docx]

**
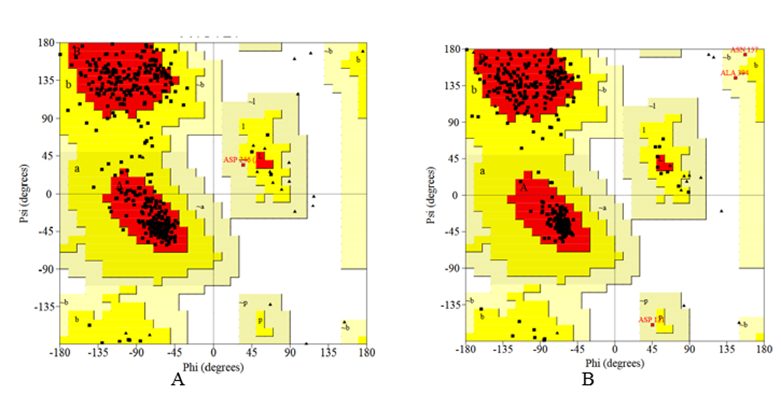
**

**S7 Fig. The Ramachandran plot and the frequency of amino acids in a different position for *C. avellana* L.**

(A) 4G0B template (B) MO323. In the picture, [A, B, L] show a very favored region for amino acids. [a, b, l, p] show the allowed zone, and [~a, ~b, ~l, ~p] show the permitted regions with ignorance.
