# Supplementary material for: Identification and in-silico characterization of taxadien-5α-ol-O-acetyltransferase (TDAT) gene in Corylus avellana L
Source: PLoS One. 2021 Aug 27;16(8):e0256704. doi: 10.1371/journal.pone.0256704 (PMC8396717; doi:10.1371/journal.pone.0256704)
Supplement: S1 Table — (Thermo Scientific (Fermentase), cat no.: K1621, USA). (DOCX) [file pone.0256704.s008.docx]

**S1 Table**. **PCR Primers sequences for *GAPCH* gene.**

| Gene | 5’Forward3’ | 5’Reverse3’ | A.T. (°C) |
| --- | --- | --- | --- |
| GAPDH | CAAGGTCATCCATGACAACTTTG | GTCCACCACCCTGTTGCTGTAG | 58 |

(Thermo Scientific (Fermentase), cat no.: K1621, USA)
