# Supplementary material for: Identification and in-silico characterization of taxadien-5α-ol-O-acetyltransferase (TDAT) gene in Corylus avellana L
Source: PLoS One. 2021 Aug 27;16(8):e0256704. doi: 10.1371/journal.pone.0256704 (PMC8396717; doi:10.1371/journal.pone.0256704)
Supplement: S2 Table — The most portable framework and the tallest of ORF are in + 1 and the nucleotide of 97 to 1398. (DOCX) [file pone.0256704.s009.docx]

**S2 Table**. **The results of ORFs^,^ online software.**

| **label** | **strand** | **Frame** | **Start** | **Stop** | **Length (nt│aa)** |
| --- | --- | --- | --- | --- | --- |
| **ORF1** | **+** | **1** | **97** | **1398** | **1302│433** |
| ORF6 | - | 3 | 1411 | 1214 | 198│65 |
| ORF7 | - | 3 | 1057 | 893 | 165│54 |
| ORF5 | - | 2 | 464 | 324 | 141│46 |
| ORF9 | - | 3 | 673 | 548 | 126│41 |
| ORF8 | - | 3 | 805 | 692 | 114│37 |
| ORF4 | - | 2 | 689 | 585 | 105│34 |
| ORF3 | - | 2 | 1046 | 945 | 102│33 |
| ORF2 | + | 3 | 120 | 209 | 90│29 |

The most portable framework and the tallest of ORF are in + 1 and the nucleotide of 97 to 1398.
