# Supplementary material for: Identification and in-silico characterization of taxadien-5α-ol-O-acetyltransferase (TDAT) gene in Corylus avellana L
Source: PLoS One. 2021 Aug 27;16(8):e0256704. doi: 10.1371/journal.pone.0256704 (PMC8396717; doi:10.1371/journal.pone.0256704)
Supplement: S1 Raw images — (PDF) [file pone.0256704.s010.pdf]

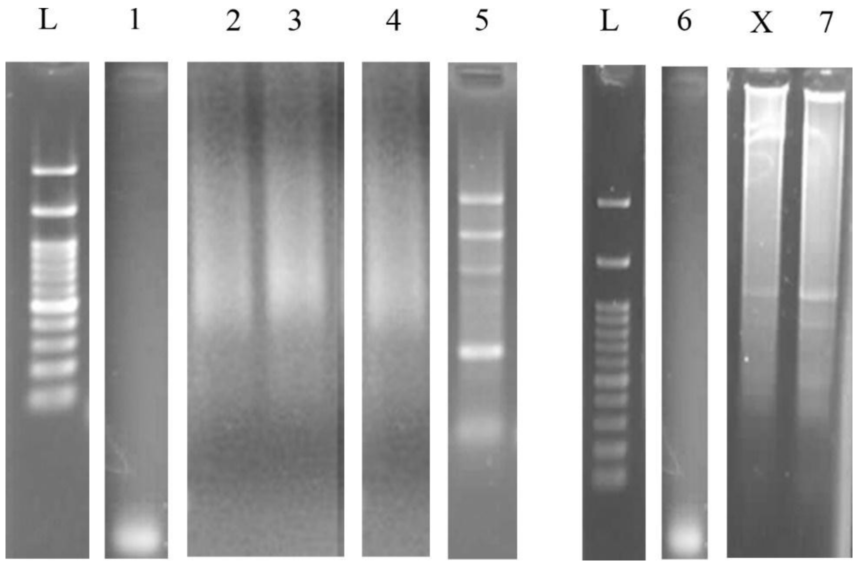

S2 Fig. Agarose (1%) gel electrophoresis.

The unadjusted image for S2 Fig is shown above. The labels on each lane are the same as the ones in S2 Fig not include lanes marked with "×". This image (S2 Fig) is in the supplementary information. Only the original cropped photos are available in the image. The cropped parts of images were identified with the blanks among the sections. The photo was captured using the VIBER Company's Quantum gel documentation imaging technology.

L: (ladder 100 bp), 1: Negative control (water), 2: RT-PCR products without any treatment (control), 3: RT-PCR product with 50 µM of MeJA, 4: RT-PCR product with 100 µM of MeJA, 5: RT-PCR product with 150 µM MeJA, 6: Negative control, 7: PCR product after PCR-purification in 150 µM of MeJA, X: not included lane. The grouping of gels which have been cropped from different gels was identified with vertical white lines.

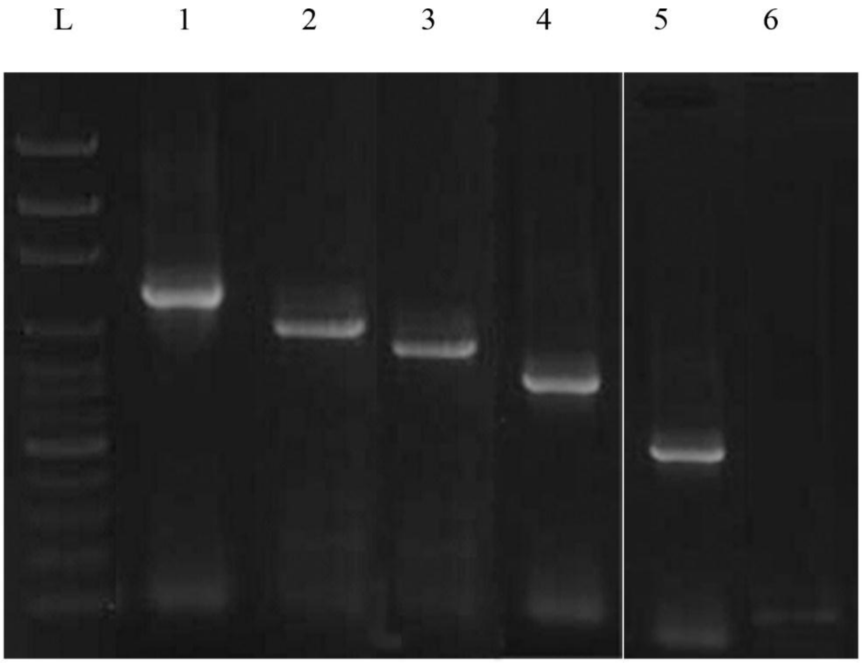

S3 Fig. Agarose (1%) gel electrophoresis of nested-PCR analysis of *C. avellana*.

The unadjusted image for S3 Fig is shown above. The labels on each lane are the same as the ones in S3 Fig. This image (S3 Fig) is in the supplementary information. The cropped parts of images were identified with the blanks among the sections. The photo was captured using the VIBER Company's Quantum gel documentation imaging technology.

Ladder (L) 100bp,1: Forward 1 + Reverse 1 (Fr_1+Rr_1), 2. Forward 1+ Reverse 2 (Fr_1+Rr_2), 3. Forward 2 + Reverse 1 (Fr_2+Rr_1), 4. Forward 2 + Reverse 2 (Fr_2+Rr_2), 5. Positive control (GAPDH primers), 6. Negative control (water).
