# Supplementary material for: Identification and in-silico characterization of taxadien-5α-ol-O-acetyltransferase (TDAT) gene in Corylus avellana L
Source: PLoS One. 2021 Aug 27;16(8):e0256704. doi: 10.1371/journal.pone.0256704 (PMC8396717; doi:10.1371/journal.pone.0256704)
Supplement: Attachment — Submitted filename: PONE-D-21-05459_reviewer.pdf [file pone.0256704.s011.pdf]

PLOS ONE

Identification and in-silico characterization of taxadien-5α-ol-O-acetyltransferase (TDAT) gene in Corylus avellana L.

--Manuscript Draft--

**Manuscript Number:**	PONE-D-21-05459
**Article Type:**	Research Article
**Full Title:**	Identification and in-silico characterization of taxadien-5α-ol-O-acetyltransferase (TDAT) gene in Corylus avellana L.
**Short Title:**	Identification of taxadien-5α-ol-O-acetyltransferase (TDAT) gene in Corylus avellana L
**Corresponding Author:**	Sara Alsadat Rahpeyma<br>Shahid Bahonar University Faculty of Agriculture<br>Kerman, Kerman IRAN, ISLAMIC REPUBLIC OF
**Keywords:**	Corylus avellana L.; * Protein structure; Paclitaxel; * taxadiene-5α-ol-O-acetyltransferase; *
**Abstract:**	Paclitaxel ® (PC) is one of the most effective and profitable anti-cancer drugs and tissue cultures of Taxus species and, most recently, hazelnut (Corylus avellana L.) represent the most promising sources of this compound. A large part of the PC biosynthetic pathway in the yew and a few steps in the hazelnut have been identified. Since understanding the biosynthetic pathway of medicinal metabolites in plants is an effective step toward their development and engineering, this study undertook to identify taxadiene-5α-ol-O-acetyltransferase (TDAT) in hazelnut. TDAT is one of the key genes involved in the third step of the PC biosynthetic pathway. In this study, the TDAT gene was isolated using the nested-PCR method and then characterized. The cotyledon-derived cell mass induced with 150 µM of methyl jasmonate (MeJA) was used to isolate RNA and synthesize the first-strand cDNA. The full-length cDNA of TDAT has a length of 1423 bp which contains 1302 bp ORF encoding 433 amino acids. The phylogenetic analysis of this gene presented high homology with its ortholog genes in Quercus suber and Juglans regia . Bioinformatics analyses were used to predict the secondary and tertiary structures of the protein. Protein structure prediction revealed that this protein may operate at the cytoplasm due to the lack of signal peptide. The homologous superfamily of the T5AT protein has two domains. The highest and lowest hydrophobicity of amino acids were found in proline 142 and lysine 56, respectively. T5AT protein fragment had 24 hydrophobic regions. The tertiary structure of this protein was designed using Modeler software (V.9.20), and its structure was verified with the results of the Verify3D (89.46%) and ERRAT (90.3061) programs. The T5AT enzyme belongs to the superfamily of the transferase, and the amino acids histidine 164, cysteine 165, leucine 166, histidine 167, and Aspartic acid 168 were located at its active site.
**Order of Authors:**	Mona Raeispour Shirazi
	Sara Alsadat Rahpeyma
	Sajad Rashidi Monfared
	Jafar Zolala
	Azadeh Lohrasbi-Nejad
**Additional Information:**	
**Question**	**Response**
**Financial Disclosure**<br><br>Enter a financial disclosure statement that describes the sources of funding for the work included in this submission. Review the submission guidelines for detailed	The author(s) received no specific funding for this work

Powered by Editorial Manager® and ProduXion Manager® from Aries Systems Corporation

requirements. View published research articles from *PLOS ONE* for specific examples.<br><br>This statement is required for submission and **will appear in the published article** if the submission is accepted. Please make sure it is accurate.<br><br>**Unfunded studies**<br>Enter: *The author(s) received no specific funding for this work.*<br><br>**Funded studies**<br>Enter a statement with the following details:<br>• Initials of the authors who received each award<br>• Grant numbers awarded to each author<br>• The full name of each funder<br>• URL of each funder website<br>• Did the sponsors or funders play any role in the study design, data collection and analysis, decision to publish, or preparation of the manuscript?<br>• **NO** - Include this sentence at the end of your statement: *The funders had no role in study design, data collection and analysis, decision to publish, or preparation of the manuscript.*<br>• **YES** - Specify the role(s) played.<br><br>* typeset	
**Competing Interests**<br><br>Use the instructions below to enter a competing interest statement for this submission. On behalf of all authors, disclose any competing interests that could be perceived to bias this work—acknowledging all financial support and any other relevant financial or non-financial competing interests.<br><br>This statement is required for submission and **will appear in the published article** if the submission is accepted. Please make sure it is accurate and that any funding sources listed in your Funding Information later in the submission form are also declared in your Financial Disclosure	The authors have declared that no competing interests exist.

statement.<br><br>View published research articles from *PLOS ONE* for specific examples.<br><br>**NO authors have competing interests**<br><br>Enter: *The authors have declared that no competing interests exist.*<br><br>**Authors with competing interests**<br><br>Enter competing interest details beginning with this statement:<br><br>*I have read the journal's policy and the authors of this manuscript have the following competing interests: [insert competing interests here]*<br><br>* typeset	
**Ethics Statement**<br><br>Enter an ethics statement for this submission. This statement is required if the study involved:<br><br>• Human participants<br>• Human specimens or tissue<br>• Vertebrate animals or cephalopods<br>• Vertebrate embryos or tissues<br>• Field research<br><br>Write "N/A" if the submission does not require an ethics statement.<br><br>General guidance is provided below. Consult the submission guidelines for detailed instructions. **Make sure that all information entered here is included in the Methods section of the manuscript.**	N/A

**Format for specific study types**<br><br>**Human Subject Research (involving human participants and/or tissue)**<br>• Give the name of the institutional review board or ethics committee that approved the study<br>• Include the approval number and/or a statement indicating approval of this research<br>• Indicate the form of consent obtained (written/oral) or the reason that consent was not obtained (e.g. the data were analyzed anonymously)<br><br>**Animal Research (involving vertebrate animals, embryos or tissues)**<br>• Provide the name of the Institutional Animal Care and Use Committee (IACUC) or other relevant ethics board that reviewed the study protocol, and indicate whether they approved this research or granted a formal waiver of ethical approval<br>• Include an approval number if one was obtained<br>• If the study involved *non-human primates*, add *additional details* about animal welfare and steps taken to ameliorate suffering<br>• If anesthesia, euthanasia, or any kind of animal sacrifice is part of the study, include briefly which substances and/or methods were applied<br><br>**Field Research**<br><br>Include the following details if this study involves the collection of plant, animal, or other materials from a natural setting:<br>• Field permit number<br>• Name of the institution or relevant body that granted permission	
**Data Availability**<br><br>Authors are required to make all data underlying the findings described fully available, without restriction, and from the time of publication. PLOS allows rare exceptions to address legal and ethical concerns. See the PLOS Data Policy and FAQ for detailed information.	Yes - all data are fully available without restriction

Powered by Editorial Manager® and ProduXion Manager® from Aries Systems Corporation

A Data Availability Statement describing where the data can be found is required at submission. Your answers to this question constitute the Data Availability Statement and **will be published in the article**, if accepted.<br><br>**Important:** Stating 'data available on request from the author' is not sufficient. If your data are only available upon request, select 'No' for the first question and explain your exceptional situation in the text box.<br><br>Do the authors confirm that all data underlying the findings described in their manuscript are fully available without restriction?	
**Describe where the data may be found in full sentences. If you are copying our sample text, replace any instances of XXX with the appropriate details.**<br><br>• If the data are **held or will be held in a public repository**, include URLs, accession numbers or DOIs. If this information will only be available after acceptance, indicate this by ticking the box below. For example: *All XXX files are available from the XXX database (accession number(s) XXX, XXX.).*<br>• If the data are all contained **within the manuscript and/or Supporting Information files**, enter the following: *All relevant data are within the manuscript and its Supporting Information files.*<br>• If neither of these applies but you are able to provide **details of access elsewhere**, with or without limitations, please do so. For example:<br><br>*Data cannot be shared publicly because of [XXX]. Data are available from the XXX Institutional Data Access / Ethics Committee (contact via XXX) for researchers who meet the criteria for access to confidential data.*<br><br>*The data underlying the results presented in the study are available from (include the name of the third party*	All relevant data are within the manuscript and its Supporting Information files.

and contact information or URL).<br>• This text is appropriate if the data are owned by a third party and authors do not have permission to share the data.<br><br>* typeset	
Additional data availability information:	Tick here if your circumstances are not covered by the questions above and you need the journal's help to make your data available.

Manuscript Click here to access/download;Manuscript;TDATplos 2.docx

Identification and *in-silico* characterization of taxadien-5α-ol-*O*-acetyltransferase (*TDAT*) gene in *Corylus avellana* L.

Mona Raeispour Shirazi[1], Sara Alsadat Rahpeyma[1]*, Sajad Rashidi Monfared[2], Jafar Zolala[1], Azadeh Lohrasbi-Nejad[1]

[1] Department of Agricultural Biotechnology, Shahid Bahonar University of Kerman, Kerman, Iran. P.O. Box: 76169-133

[2] Department of Biotechnology, Faculty of Agriculture, Tarbiat Modares University, Tehran, Iran. P.O. Box: 14115-336

*Corresponding author:
E-mail: s.rahpeyma@uk.ac.ir, 0000 0001 5518 3703 (ORCID)

Abstract

Paclitaxel® (PC) is one of the most effective and profitable anti-cancer drugs and tissue cultures of *Taxus* species and, most recently, hazelnut (*Corylus avellana* L.) represent the most promising sources of this compound. A large part of the PC biosynthetic pathway in the yew and a few steps in the hazelnut have been identified. Since understanding the biosynthetic pathway of medicinal metabolites in plants is an effective step toward their development and engineering, this study undertook to identify *taxadiene-5α-ol-O-acetyltransferase* (*TDAT*) in hazelnut. *TDAT* is one of the key genes involved in the third step of the PC biosynthetic pathway. In this study, the *TDAT* gene was isolated using the nested-PCR method and then characterized. The cotyledon-derived cell mass induced with 150 µM of methyl jasmonate (MeJA) was used to isolate RNA and synthesize the first-strand cDNA. The full-length cDNA of *TDAT* has a length of 1423 bp which contains 1302 bp ORF encoding 433 amino acids. The phylogenetic analysis of this gene presented high homology with its ortholog genes in *Quercus suber* and *Juglans regia*. Bioinformatics analyses were used to predict the secondary and tertiary structures of the protein. Protein structure prediction revealed that this protein may operate at the cytoplasm due to the lack of signal peptide. The homologous superfamily of the T5AT protein has two domains. The highest and lowest hydrophobicity of amino acids were found in proline 142 and lysine 56, respectively. T5AT protein fragment had 24 hydrophobic regions. The tertiary structure of this protein was designed using Modeler software (V.9.20), and its structure was verified with the results of the Verify3D (89.46%) and ERRAT (90.3061) programs. The T5AT enzyme belongs to the superfamily of the transferase, and t[illegible]mino acids histidine 164, cysteine 165, leucine 166, histidine 167, and Aspartic acid 168 were located at its active site.

Keywords *Corylus avellana* L. · Paclitaxel · *taxadiene-5α-ol-O-acetyltransferase* · Protein structure

Introduction

Cancer is known as a leading cause of death worldwide, and it is expected that the number of cancer patients will increase to more than 22 million cases in the next 20 years [1]. Consequently, the demand for anticancer drugs is growing rapidly. Paclitaxel (PC), sold under the brand name Taxol, is a chemotherapy medication used to treat various types of cancer such as ovarian and breast cancers as well as AIDS-related Kaposi's Scarcorna [2-4]. PC has a unique effect on cancer cells compared to other similar compounds and it inhibits cell proliferation by binding to microtubules. This compound also promotes the stabilization of microtubules at the G2-M phase of the cell cycle [3, 5]. PC is a diterpenoid compound that was first extracted from the western yew tree (*Taxus brevifolia*). In particular, Hazelnut (*Corylus avellana* L.) [6], some microorganisms like yew endophytic fungi, and hazelnut endophytic fungi [7, 8] are the PC's sources. All natural sources produce low levels of PC [9-11]. Although PC can be synthesized through total chemical synthesis, it is found to be time-consuming, expensive, and low-yielding due to the complex chemical structure of this compound [12, 13]. Hence, in response to the increasing demand for the supplies of PC, new alternative approaches are required to be developed. It has been well investigated that *Taxus* and *C. avellana* L. cell cultures are promising sources of PC production [4, 14, 15]. Metabolic engineering introduces rational changes in the genetic makeup of an organism to alter or improve its metabolic profile, and consequently to develop new "non-natural" products. The control of these complex biosynthetic processes has been enabled by the understanding of the metabolic pathways and advances in molecular biology techniques [16]. However, the amount of PC produced by tissue and cell culture of *C. avellana* is low. The deep and detailed understanding of the PC biosynthetic pathway in hazelnut, especially those genes encoding rate-limiting enzymes and the enzymes catalyzing these reactions, as long with developments in metabolic engineering, account for developing practical and effective biotechnological methods that result in a considerable increase in the amount of PC produced in hazelnut [17, 18].

The PC biosynthesis pathway contains 19 enzymatic steps. This pathway starts from the universal precursor of a diterpenoid called geranylgeranyl diphosphate (GGPP). GGPP is created from farnesyl diphosphate (FPP) and isopentenyl diphosphate (IPP) by GGPP synthase (GGPPS)[19]. A few PC biosynthetic genes have been identified in hazelnut, such as *GGPPS* (Gene Bank Accession No: EF 206343) and *CgHMGR* (Gene Bank Accession No: EF553534) [18, 20].

The initial main precursor PC biosynthesis pathway is taxa-4(5), 11(12)-diene. This precursor is catalyzed by taxadien synthase (TS) from GGPP [21]. Taxadien is hydroxylated at the C-5 position and produces taxa-4(20),11(12)-dien-5α-ol [22]. The first acetyl transferase in the PC biosynthetic pathway is taxadient-5α-ol-*O*-acetyl transferase (T5AT) which converts taxa-4(20),11(12)-dien-5α-ol to taxa-4(20), 11(12)-dien-5α-yl-acetate. T5AT acrylates taxa-4(20), 11(12)-dien-5α-ol at the C-5 position in the presence of acetyl CoA [19]. This reaction is important due to the presence of an important branch in this position. T5AT competes with taxadien-13α-hydroxylase (TαH). TαH like T5AT converts taxa-4(20), 11(12)-diene-5α-ol to taxa-4(20), 11(12) diene 5α, 13α-diol, but its next steps are unknown. Thus, the alternative branch in this pathway conquers *taxadien-5α-ol-O-acetyltransferase* (*TDAT*) could be an important target for metabolic engineering [23, 24].

In this article, we present the result of a novel study on th[illegible]olation and characterization of *TDAT* from the methyl jasmonate (MeJA)-induced cell suspension of hazelnut.

Materials and methods

In vitro cell culture chemical and biological reagents

In this research, the plant materials were hazelnut (*C. avellana* L.) cotyledon-derived calli, and cell suspensions. For this purpose, hazelnut seeds were provided from Gilan Province of Iran, (37.1378° N, 50.2836° E). Friable calli and cell suspensions were obtained according to the immobilizing cells method[25]. Briefly, the first step was sterilization of the seeds by sodium hypochlorite (5.25 %) for 25 min, ethanol 70 % (v/v) for two min, sodium hypochlorite (25 min) again, and finally sterile water (3 x 5 min). The induced white calli of seed cotyledons in MS medium supplemented with 0.2 mg l^{-1} 6-benzylaminopurine (BA) and 2 mg l^{-1} 2, 4-dichlorophenoxy acid (2.4-D) were suspended in liquid media with the same composition. The cell mass obtained through several subcultures in liquid media was immobilized in solid media and produced friable and fast-growing calli. These calli were applied for subsequent experiments and the establishment of the next suspension. Culture medium salts and vitamins were provided by the Merck (Kenilworth, New Jersey, USA) pharmaceutical company and other reagents including plant hormones and elicitors were provided by the Sigma (St. Louis, Missouri, USA) chemical company.

Elicitor treatment of cell suspension

Frequent subcultured (at least 4 times) and homogenized cell suspensions were used for the elicitor treatment. The stock solution of MeJA was made by solving it in 0.1 % ethanol and water and then sterilizing it by filtering it through 0.22 µm filters. The cell suspension was treated on the seventh day of its cultivation (start of stationary growth phase) with various concentrations of MeJA (0, 50, 100, 150 µM) with the three replicates. In the case of control cultures (0 µM MeJA), 10 µL filter-sterilized 0.1 % ethanol were added. The elicited cells were harvested after 72 h [26]. Subsequently, the harvested cells were kept in liquid nitrogen until they were used for the next molecular studies.

EST assembly strategy for identification of TDAT

The *TDAT* coding DNA sequence (CDS) was obtained from *C. avellana* Expressed Sequence Tags (EST) library (http:// www.ncbi.nlm.nih.gov/dbEST/). In brief, ESTs were downloaded from several RNA sequence projects (SRA) of hazelnut and used to build up consensus sequences. The EST sequences of *TDAT* genes were achieved from similar orthologous sequences in other plants utilizing offline BLAST software. These EST sequences were assembled through the "align-then assembles" approach using the Codon Code Aligner V.6.0.2 software (Codon Code Corporation, USA). Then, a *TDAT* consensus sequence was created using the assembling operation and then emitting different ESTs. Accordingly, the sequenced was cleaned up and a consensus sequence was created using Codon Code Aligner. This sequence was evaluated for searching the open reading frame (ORF) and related protein using the ORF finder (http//www.ncbi.nlm.nih.gov/ovffinder).
BLASTp (http/blast.ncbi.nlm.nih.gov/blast) was carried out to confirm the ORF and protein of the *TDAT* gene. Eventually, gene-specific primers were designed for PCR-amplification of the *TDAT* gene based on bioinformatics analysis. Primer premier (V.6.0) was used to design the primers based on the full ORF of the cDNA consensus sequence (Table 1). Interaction and characteristics of primers were investigated and confirmed with the oligo analyzer (http://eu.idtdan.com/cak/analyzer) V.3.1 program.

Table 1. PCR Primers sequences for *TDAT* gene

Position	Primer	Sequences
Outside ORF	Forward .1	5’-GGGGGACAATCTCAAGTTCATT-3’
Outside ORF	Reverse .1	5’-GTGGACCAAATTGAACGTACACC-3’
Inside ORF	Forward .2	5’-CTGACAATCAGCTCAGAAGGAAAG-3’
Inside ORF	Reverse .2	5’-CTAGACCAAGTTGTGACCAGTAG-3’

Extraction of RNA and cDNA synthesis

Total RNA was extracted from MeJA treated hazelnut cells (calli) with a Total RNA isolation kit, DENAZ II ASIA, (cat No.: S-1010, Iran). The concentration and quality of the extracted RNA were analyzed using a Nanodrop spectrophotometer (OneC, Thermo Scientific, USA) and confirmed with agarose gel electrophoresis. Genomic DNA content was removed from the extracted RNA by RNase-free DNAse I (Thermo Scientific (Fermentase), cat no.: ENo521, USA). The first-strand cDNA synthesis reaction was accomplished using a Revert Aid first-strand cDNA synthesis kit (Thermo Scientific (Fermentase), cat no.: K1621, USA).

RT-PCR analysis and gene isolation with Nested-PCR

Because gene identification and isolation from cDNA depends on its expression, the cell suspension elicited with MeJA was used for RNA extraction and cDNA synthesis. Various MeJA concentrations were used to induce *TDAT* expression, and the induced cell mass with 150 μL of MeJA was used for the subsequent RT-PCR and nested-PCR. *TDAT* gene expression was investigated using RT-PCR. Nested-PCR was also performed to confirm the chara[illegible]ized ORF. Nested primers were designed in different situations of the consensus sequence. They would amplify overlap fragments to approve the ORF specification (Fig. 1). The RT-PCR experiment was operated using forward-1 and reverse-1 primers (Table 1). These primers amplified a fragment with a length of 1423 bp and an ORF of 1302 bp. The following reagents were used for RT-PCR and nested-PCR: 1 μL DNase-free water, 1 μL forward primer (10 pmol μL^{-1}), 1 μL reverse primer (10 pmol μL^{-1}), 2 μL cDNA (100-200 ng μL^{-1}), and 10 μL Tag DNA polymerase Mix Red – 1.5 mμ $Mgcl_2$ (Ampliqone, cat no.: A150303, Denmark). A volume of 20 μL of each reaction was used for PCR. The RT-PCR program, as well as nested-PCR, were as follows: pre-denaturing cDNA at 94 °C for 1 min, 34 cycles for denaturing at 94 °C within 30 s, annealing at 52± 2 °C during 35 s, and 85 s of extension at 72 °C. After the last cycle, the amplification was extended at 72 °C for 5 min. Annealing at 58 °C during 35 s was set for the RT-PCR program with GAPDH primers.

PCR purification was done using Ron’s Gel Extraction Kit (BIGRON, cat no.: 802501) for 1423 bp. The cleaned PCR products were subjected to sequencing (Bioneer, South Korea). The nested–PCR amplification and full-length cDNA sequencing were repeated four times.

Fig. 1 The situation of cDNA full length and nested-PCR primers comprising *TDAT* gene. The arrows denote the primers and the green rectangles illustrated nested-PCR products for each primer. Rr: Reverse; Fr: Forward.

Bioinformatics analysis and Modeling of the T5AT protein

The results of cDNA sequencing were analyzed using Chromas V.1.14 software. Overlapping sequences were edited using the CLC sequencing V.6.1 program, and the final sequence was used for subsequent studies. Protein sequences that identified with more than 70% of the coding region of consensus sequences from different species were chosen for sequence alignment and phylogenic analysis. The relationship between T5AT and downloaded proteins from BLASTp was determined using the web-based Clustal Omega software (http://www.ebi.ac.uk/tools/msa/clustal/), and the maximum likelihood using the MEGA V.7.0. A Dayhoff model was used to build the phylogenetic construction [27]. To calculate bootstrap (BS) values, 1000 iterations were used. The previously aligned nucleotide sequences of 15 geneses were used to draw the evolutionary tree of the *TDAT* gene, based on the branch-site model, by using the EasyCodeML software, a part of the PAML package [28]. The branches' length and Omega (ω) values were evaluated using Nei & Gojobori method [29] and Bayes Empirical Bayes (BEB) [30] analysis.

Secondary structure prediction of the T5AT protein

The secondary structure of the T5AT protein was predicted and assessed using the software. The conserved area of the T5AT gene was identified using the alignment of ortholog sequences for more precision and confidence. Interpro (http://www.ebi.ac.uk/interpro) was used to represent the domains of T5AT. The PSORT-program (www.genscript.com/tools/wol:psort) was used to determine the functional location of the T5AT protein. The position of each amino acid in the secondary structure of the protein was determined using the online PORTER (http:distillused.ie/porter) and PSIPRED (bioinf.cs.uc/ac.uk/psipred) programs. The protein storage site and gene ontology of T5AT were performed using PREDICT NLS (http://www.ppredictprotein.org). This program showed the connection between amino acids and other proteins and their molecular functions. The Signal Peptide program (http:www.cbs.tdu.dk/service/signal/) was performed to predict the signal sequence of T5AT. Epestifind software (emboss.bioinformatics.nl/cgi-bin/emboss/epestifind/) was used to rapidly identify the PEST motif (proline (P), one aspartate (D), glutamate (E), and at least one serine (S), or threonine (T)). Protparam (http:web.expasy.org/protparam) and Protscale (http://web.expasy.org/protscale) computed various physicochemical properties such as molecular weight, theoretical PI, amino acids composition, instability index, and hydrophobicity scales [31, 32].

T5AT tertiary structure prediction

The tertiary structure of the T5AT protein was assessed through the program herein described. The amino acid sequence associated with the taxadien-5α-ol-*O*-acetyltransferase enzyme was prepared from the protein sequence database of NCBI with AAB41811 code. The T5AT sequence was compared with the sequence of proteins available in the protein database (PDB) to find the appropriate template creating its tertiary structure. The template protein crystallography file (PDB ID: 4g0b) was received from a protein data bank. Modeler software (V.9.20) was used to generate 500 structural models of the T5AT protein. Verify3D and ERRAT compared the similarity of the simulated structure with the template. Procheck and Prosa software was used to calculate the quality of the space chemistry models. Procheck was used to evaluate the accuracy of the protein structure and the simulated models. For this purpose, parameters such as the quality of the Ramachandran plot were examined [33]. Finally, the similarity of the 3D-structure of the template with the selected model was

estimated by measuring the root mean squared deviation (RMSD) using Swiss-Pdb Viewer (SPDBV) software (version 4.1; http://spdbv.vital-it.ch/).

Results

Elicitor treatment of cell suspension and RT-PCR analysis

In the primary studies, no gene (*TDAT*) expression was observed in the control (none elicited) calli or cell suspensions (Fig. 2). MeJA elicitor treatments (0, 50, 100, and 150 µM) were then used to induce *TDAT* gene expression. *TDAT* gene expression at low MeJA concentrations (0, 50, and 100 µM) was such that it could not be detected. However, *TDAT* expression was induced at 150 µM of MeJA only (Fig. 2). Accordingly, 150 µM of MeJA-induced cells were used for RNA extraction and RT-PCR preparation. At this concentration, PCR purification was done using Ron's Gel Extraction Kit (BIGRON, cat No.: 802501) for 1423 bp and was delivered for sequencing.

Fig. 2. Agarose (1%) gel electrophoresis (**a**) evaluation of RT-PCR product of the different concentration of MeJA induced cells; L: (ladder 100 bp), 1: Negative control (water), 2: RT-PCR products without any treatment (control), 3: RT-PCR product with 50 µM of MeJA, 4: RT-PCR product with 100 µM of MeJA, 5. RT-PCR product with 150 µM MeJA. (**b**) evaluation of PCR purification. 6. Negative control. 7. PCR product after PCR-purification in 150 µM of MeJA. The grouping of gels which have been cropped from different gels was identified with vertical white lines.

Identification and characterization of the TDAT gene with EST assembly and Nested-PCR

First, a consensus sequence of *TDAT* was made of 152 ESTs. The ORFs and related proteins of the consensus sequence were evaluated with ORF finder and confirmed with BLAST(P). Nested-PCR was performed as a supplementary experiment to amplify the full-length of cDNA of the *TDAT* from C. avellana and merely to further confirm the results of the bioinformatics analysis. It amplified the fragments including the coding regions of *TDAT* with the designed primers (Supplementary Figure S1). The results of nested-PCR were precisely the same as what was anticipated from the bioinformatics analysis. The cDNA was 1423 bp and, according to ORF finder, it was included in 1302 bp encoding 433 amino acids (Table S1) (submitted to GeneBank as accession number: *TDAT* (*TAT*): MF134435).

Bioinformatics analysis and Modeling of T5AT protein

To draw the phylogenic tree and protected regions, the alignment of amino acids was carried out with Clustal Omega software. The HXXXDG motif is very stable and highly conserved. Its amino acid position is 164 to 169 of *C. avellana* (Fig. 3).

The results of phylogenetic analyses suggested that the *TDAT* gene in *C. avellana* is more closely related to *HHT* in *Quercus suber* and it belongs to the same order as *Quercus suber* and *Juglans regia* (Fig. 4).

Fig. 3 Multiple sequence alignment amino acids of *TDAT* gene with other plants. This software indicated HXXXDG motif.

Fig. 4 Phylogenetic analysis of identified *TDAT* of *C. avellana* with *HHT* to another plant that had high identity

One of the statistical parameters to genetically evaluate the evolution process is Omega (ω) value, the rate ratio of nonsynonymous to synonymous substitutions (d_N/d_S). ω=1 suggests neutral expectation; ω< 1 indicates negative (purifying) selection; while ω> 1 shows positive (diversifying) selection [29]. As shown in Supplementary Figure S2, the species evaluated are divided into three distinct categories according to the evolution of the *TDAT* gene. This gene has the same ancestor in *Corylus*, *Quercus*, and *Juglans*. The assessment of nucleotide changes that alter the amino acids (d_N) relative to nucleotide changes that did not affect the resulting amino acid is a practical and highly efficient method for detecting the process of natural selection during gene evolution. According to the results of the obtained matrix from the Nei & Gojobori method, d_N, d_S and ω were 0.0838, 0.4804, and 0.1745, respectively, for *C. avellana* L., indicating a purifying selection for the *TDAT* gene. Several statistical methods based on codon substitution models have been developed to identify positives selection for a branch in a phylogeny. One of the simulation studies in the phylogenetic analysis by maximum likelihood (PAML), using the maximum-likelihood methods is Bayes Empirical Bayes (BEB) analysis. It has been inferred from the results that Thr106 in *C. avellana*, approximately 98%, had been influenced under positive selection. The probability of changes in amino acids Gly24, Phe32, Phe34, Pro46, Lys47, Gly50, Ala79, Gly161, Gln206, Val242, Ser307, and Pro376 was 50% to 79%. The length of the branches shows the number of nucleotide substitutions per codon (Supplementary Figure S2). As a result, most nucleotide changes were observed for *Juglans* (0.27151) in comparison with *Corylus* (0.26287) and *Quercus* (0.22245).

Secondary structure prediction of T5AT protein

The results of protein domains analysis, performed using the InterPro database and tools, predicted that the family of the T5AT protein would belong to transferases. The homologous superfamily of the T5AT protein has two domains. The position of the first domain was found in 7 to 216 amino acids and the second domain was located in 220 to 432 amino acids. The molecular function of this enzyme was foreseen as the transmission of the Acyl group using PREDICT. The NLS analyzed the cytoplasmic localization of this protein. This software confirmed the InterPro predicted molecular function. All protein binding sites (PBS) which catalysis the activity and the biological process of this protein in the biosynthesis of the metabolites, were shown by PREDICT NLS (Fig. 5). They are located in the regions of 1-4, 53-56, 116-120, 212-213, 397-397, and 408-410 amino acids.

Fig. 5 The protein binding sites (PBS) showed by PREDICT NLS. All indicated parts with red diamonds are the PBS. (The regions are 1 to 4, 53 to 56, 116 to 120, 212 to 213, 397 to 397, and 408 to 410).

Based on the Signal P program, no signal peptide was determined for the T5AT protein. According to Porter's data prediction, the T5AT protein is formed through the contribution of 29.6 % Helix, 26.1 % extended or Beta strand, and 44.34% coils (Fig. 6). The secondary structure of the T5AT protein was confirmed with the same result of the PSIPRED program with the line confidence of 0 to 9 digits. The PSIPRED prediction of the location of the T5AT protein was performed in 6 positions with different scores. The activity site of this protein in the plant cell was predicted to be in the cytoplasm (50%), nucleus (21.5%), mitochondrion (7.1%), peroxisome (7.1%), endoplasmic reticulum (7.1%) and chloroplast (7.1%).

Fig. 6 The secondary structure of T5AT protein prediction with PORTER program. Query-length: 433. H= Helix. E= strand. C = Coil. B = much buried. b = somewhat buried. e = somewhat exposed. E =very exposed.

Epestifind software was used to find the PEST motif as a potential proteolytic cleavage site. Altogether 4 PEST motifs were identified in the T5AT protein from position 1 to 433. A potential PEST motif with 14 amino acids was determined among the positions of 14 to 29, and three poor PEST motifs were considered among the amino acids of 136 to 150, 319 to 337, and 378 to 398 (Fig. 7).

Fig. 7 The position of the PEST motif was detected with Epestifind software.

Using Protparam software, the molecular weight of the TDAT protein and theoretical pI were estimated to be 48017.62 Da and 6.43, respectively (Table 2). The result of Protscale analysis showed that T5AT has 51 positive electric charges and 53 negative electric charges, due to the presence of lysine/ arginine and aspartate/glutamate. The details of the abundance of amino acids participating in the T5AT structure are summarized in Table 2. The molecular formula of this protein was registered as $C_{2171}H_{3434}N_{560}O_{628}S_{18}$. The instability index for this protein was 37.94, which indicated the protein's stability. GRAVY (grand average of hydropathy) was -0.090. Furthermore, the results at the Kyte & Doolittle plot (Fig. 8) ,as well as tertiary structure, showed that T5AT protein fragments had 24 hydrophobic regions with amino acids 33-35, 41-47, 64-66, 68-77, 81-83, 87-89, 91-107, 124-132, 135-150, 152-163, 168-176, 189-191, 194-197, 240-243, 264-270, 288-292, 308-317, 328-339, 352-355, 363-373, 378-379, 382-393, 397-405, and 414-429. The most hydrophobic residue (2,033) and the lowest hydrophobicity residue (-2,644) were Pro 142 and Lys 56, respectively.

Fig. 8 The results of Kyte and Doolittle diagram about the T5AT protein containing 24 hydrophobic regions

Table 2 A glance at the prediction of simple physicochemical specifications (amino acids composition) of the T5AT protein. The amount of amino acid leucine (Leu) in the T5AT protein structure was the most.

Amino acids	numbers	Amount (%)
Ala (a)	30	6.9%
Arg (R)	16	3.7%
Asn (N)	12	2.8%
Asp (D)	21	4.8%
Cys (C)	9	2.1%
Gln (Q)	14	3.2%
Glu (E)	32	7.4%
Gly (G)	29	6.7%
His (H)	7	1.6%
Iis (I)	25	5.8%
Leu (L)	42	9.7%
Lys (K)	35	8.1%
Met (M)	9	2.1%
Phe (F)	25	5.8%
Pro (P)	27	6.2%
Ser (S)	27	6.2%
Thr (T)	26	6.0%
Trp (w)	4	0.9%
Tyr (Y)	9	2.1%
Val (V)	34	7.9%
Pyl (O)	0	0.0%
Sec (U)	0	0.0%

Number of amino acids: 433
Molecular weight: 48017.62
Theoretical pI: 6.43

3D-structure of the T5AT protein

Primarily, similar sequences that were identified most closely with the T5AT sequence were obtained using the protein BLAST database. The hydroxycinnamoyl-COA shikimate/quinate hydroxycinnamoyl transferase enzyme, it's the 3D-structure (PDB code; 4G0B) of which was identified by Lallemand *et al.* [34], was considered as a template for the T5AT protein. The sequence identity between the two proteins was determined to be 35%. Five hundred structural models for T5AT were created using Modeller software. Validation tests evaluated the quality of each model, and the most similar model was considered as the tertiary structure of the T5AT protein under the name of MO323. The results of Ramachandran plots (Fig. S3) were summarized in Table 3. Veritably, 87.0% and 88.0% of the total amino acids were located in the most favored regions for the template and MO323, respectively. The slight difference between the obtained values can be explained by the position of amino acids in specific regions of the Ramachandran plot, implying that the 3D-structure of MO323

is highly similar to the template. The overall quality of the MO323 was checked by comparing Z-scores in Prosa software. The Z-score calculates the entire energy of the structure and shows the degree of consistency between the sequence and the tertiary structure of the model [35]. This value was found to be -9.94 and -8.72 for the pattern and MO323, respectively. The negative values represent the accuracy of the simulated structure; accordingly, there is a high similarity between MO323 and the template. As shown in Fig. 9, the structure of MO323 was located in the position corresponding with the X-ray structures. Thus, the obtained model is sufficiently reliable and is empirically close to the 3D-structure of the pattern. Verify3D calculations showed that 60.51% of the amino acids belonging to MO323 had a score of higher than 0.2. This value was 89.46% for the template. This parameter determines the compatibility of the 3D-structure of the protein with its 1D-structure. Based on the obtained score, the accuracy of the simulated structure and its high quality can be inferred. ERRAT values equaled 75.8 and 90.5 for MO323 and the template, respectively. This parameter examines non-bonded interactions between different atom types and the performance rates of 9 amino acids versus 9 other amino acids through statistical comparison with the most similar structures. Based on the results, MO323 was selected from 500 models as the T5AT 3D-structure. The RMSD values of the template and the model (based on backbone and α-carbon) were 0.79 and 0.81 angstroms, respectively. The slight differences in RMSD values between the two proteins demonstrate their structural similarity. The 3D-structures belonging to the template and MO323 were merged, and the overall similarity and minor differences between them were examined with YASARA software. The result showed that the topology of two proteins' folding is significantly similar, although minor differences between the model and the pattern were seen (Fig. 10). Differences are more specified in the turn and coil regions. As shown in Fig. 11a, the T5AT enzyme in its tertiary structure has 15 beta-strands and 9 alpha-helices. Beta-strands were determined in the amino acids with 12-21, 45-51, 30-32, 82-86, 89-95, 100-107, 143-151, 154-163, 231-239, 281-294, 310-318, 368-372, 388-392, 399-405, and 412-420. Alpha-helices were defined in amino acids with 35-40, 61-72, 168-184, 240-249, 261-278, 319-326, 328-341, 343-355, and 423-432. The T5AT enzyme belongs to the transferase superfamily that is commonly found in plants and fungi. The HXXXD motif forms part of the active site of the enzymes in this superfamily [36]. This motif in the T5AT at positions 164 to 168 of the protein structure contains the His-Cys-Leu-His-Asp amino acid sequence that forms a turn between the eighth beta-strand and the tertiary alpha-helix (Fig. 11b).

Table 3 Related parameters for confirmation of the simulated structure and its similarity to the pattern.

Model	Procheck				Verify 3D	Errat
	most favored regions	additional allowed regions	generously allowed regions	disallowed regions		
**4G0B**	87.0%	12.7%	0.3%	0.0%	89.46%	90.3061
**MO323**	88.8%	10.4%	0.8%	0.0%	60.51%	75.5882

Fig. 9 Z-score values for (**a**) the template, (**b**) the simulated model (MO323). Both structural-models located in the position of X-ray structures

Fig. 10 Comparison of the surface of the 3D-structure belongs to the template and MO323 (red and yellow, respectively), differences of structures are relative to turn and coil regions

Fig. 11 3D-structure of the T5AT enzyme presented by YASARA program. (**a**) Beta-strands and alpha-helixes are yellow and blue, respectively. This model has 15 beta-strands and 9 alpha-helices; (**b**) HXXXD motif in T5AT enzyme contains His 164, Cys 165, Leu 166, His 167 and Asp168

Discussion

In this study, *taxadien-5α-ol-O-acetyltransferase* (*TDAT* or *TAT*) ortholog was detected and identified from *C. avellana* L. using the experimental and *in-silico* analysis. *C. avellana* is known as a candidate for a PC production source [37]. The full-length cDNA encoding *TDAT* from *C. avellana* (gene accession number: MF134435) was 1423 bp, similar in size to *omega-hydroxypalmitate o-feruloyl transferase* (*HHT*). The cDNA of *TDAT* contains a 1302 bp ORF encoding a protein of 433 amino acids. Drawing the phylogenetic tree indicated a high degree of identity between *TDAT* and *HHT*. *Corylus*, *Quercus suber*, *Juglans regia*, *Morus notabilis*, and *Ziziphus jujube* were in a branch of the phylogenetic tree. It should be noted that each of these plants belongs to different families, indicating this gene was protected in distant families. Investigation of the genetic evolution of *TADT* showed that Thr106 had a 98% positive change compared to other species. The coding sequence of this amino acid for *Corylus* is ACA, while other species in this position have GAA (12 cases) and GAG (2 cases) for Glu. Thr106 in the 3D-structure of T5AT protein has been placed in the beta structure and away from the active site of the enzyme. So alteration of this amino acid probably has little effect on the active site. A reviewed 3D-structure of this enzyme showed that Gly24, Phe32, Phe34, Pro46, Lys47, Ala79, Gly161, Ser307, and Pro376 are close to the active site, and alteration of these amino acids may affect the function of these enzymes.

The *taxadien-5α-ol-O-acetyltransferase* gene has been identified in the yew, another main source of PC. This gene, called *TmTAT*, was known in *T. media*. The ORF of the *TmTAT* gene was 1317 bp encoding 439 amino acids [38]. The molecular weight of the T5AT protein was 48.17 KDa. The theoretical pI of T5AT was 643, and the instability index for this protein was 37.94. The result of BLASTp indicated that the T5AT protein was closely similar to the HHT protein in *Juglans regia* and *Ziziphus jujube*. The isoelectric point of HHT in *J. regia*

and *Z. jujube* was 5.83 and 5.66, respectively. The instability index of HHT in *J. regia* and *Z. jujube* was calculated as 39.31 and 35.75, respectively. The molecular weight of the HHT protein was very close to that of the T5AT protein - approximately 48 KDa. The HHT protein had 432 amino acids in both plants.

To investigate the evolutionary relations between the transferase in *Taxus* and *Corylus*, a phylogenetic tree was constructed based on the amino acid sequences of TmTAT and transferase from other *Taxus* species. The results highlighted that TmTATs, (T5ATs) from *T. chinesis* and *T. cuspidata* were grouped into the cluster with the shortest distance from the T5AT of *C. avellana* while the TmTAT from *T. canadensis* was placed to a cluster farther away from *C. avellana*. Moreover, the T5ATs in *C. avellana* and *Taxus* species were found to be dissimilar [17]. The *taxadien-5a-ol-O-acetyl transferase* ORF in *T. chinensis* had 1275 nucleotides that encodes 425 amino acids. The protein had an isoelectric point of 5.39 and a molecular weight of 47KDa [39]. The molecular weight of the TmTAT was 49 KDa, and its instability index was 35, similar to the other *Taxus* species. On close scrutiny, *TmTAT* was strongly expressed in the leaves, weakly expressed in the stems, and had no expression in the fruits of *T. media*. A detailed look at this tissue expression pattern revealed that this is a tissue-specific gene [17]. The T5AT protein had two domains as did the HHT protein in *Quercus suber*. While, the position of the first domain of the two proteins was 7 to 216, but the position of the second domain was found to be different. in contrast, the second domain of HHT was located in amino acids 221 to 431, which differed in the position of just one amino acid in T5AT (220 to 430). However, these second domains indicated that T5AT and HHT belong to the superfamily of transferase enzymes [17, 39].

The T5AT and HHT proteins, as well as most of the acetyltransferase enzymes, have a functional HXXXDG motif. This motif was present in the position of amino acid 164 [36]. In *T. chinensis*, this protein has a functional HXXXDG motif with the function of transferring acetyl from the precursor of the CoA-acetyl and additionally, two other domains in positions 7 to 214 and 223 to 431 [39]. Based on the NCBI database, the TmTAT protein has a second domain belonging to the family of the transferase, a functional HXXXDG motif, which plays a role in the transmission of the acetyl group, and also two domains in the position of amino acids 8 to 214 and 223 to 431 [17]. It was revealed that the HXXXD motif is probably part of the activity site [36]. The previous studies indicated a lack of signal peptide in T5AT, HHT, and TmTAT proteins; therefore, these proteins probably act in the cytoplasm. The biological process of the T5AT protein was predicated in the biosynthesis of metabolites, transferase activity, and the transmission of an acetyl group. The molecular function of taxadien-5a-ol-*O*-acetyltransferase (T5AT) was identified as the transition acetyl group from acetyl-CoA and production of the T5AT enzyme in *T. cuspidate*. This process was the biological process in the PC biosynthesis [23]. Furthermore, the molecular function and biological process of the TmTAT enzyme have been reported to be the same as those of T5AT in *T. cuspidate* [17]. In this study, 29.6% Helix, 26.1% Extended, and 44.34% Coils were observed in the secondary structure of T5AT with 433 amino acids, while 37% Helix, 26% Beta strand or Extended, and 37% Coils contributed to the formation of the TmTAT protein [39]. The pattern of the T5AT protein in drawing the three-dimensional structure was HOST in *Coffea canephora* (PDB Id: 4g0b). It was confirmed that His-153 in HCT is an activity site, as the His-153-ALa mutation had no enzymatic activity as a catalytic enzyme. The His-153 in HCT from the conserved HXXXD motif was confirmed as a catalytic residue by mutagenesis [40]. Therefore, His-164 in the T5AT protein (*C. avellana*) is most likely an activity site. The activity sites in the T5AT in *T. cuspidate* were H-164 and D-373. As a result, it is most likely that D-373 is another activity site in the T5AT (*C. avellana*). ALa-156-Ser mutants produced 4-fold more diCQAs. Ser

has OH and it is a polar amino acid. Its mutation increased the activity of the enzyme; thus, it is predicted that His-167 enhances enzyme activity, because His has O^+ and is a polar amino acid, too [34]. The HST protein pattern in *Arabidopsis thaliana* (PDB Id: 5kjs) was used to draw the tertiary structure of the HCT protein in *C. canephora*. The activity sites were His-153 and Asp-380 in HST and His-153 and Asp-381 in HCT. The conserved motif in the two proteins was HHAADG. The conserved motif in the position of 164 of T5AT might be the activity site, because of the high identity between our sequence and that of HCT. The superfamily of these proteins were transferase, and their subfamilies were HST, HCT, and T5AT [19, 34].

Conclusion

In this study, *taxadiene-5α-ol-O-acetyltransferase* (*TDAT*) which catalyzed the third step of PC biosynthesis in *C. avellana*, was identified and characterized using bioinformatics analysis. The use of an exact concentration of elicitor to induce gene expression as well as the use of the EST library and bioinformatics studies were the key reasons for our success in identifying this gene. It can be deduced that the taxadien-5α-ol-*O*-acetyltransferase enzyme has undergone many changes during development. This enzyme differs significantly from the T5AT enzyme in *Taxus*, but the activity site and conserved motif were the same in the two plants and similar performances in different plants. This gene was in the primary PC biosynthetic pathway. T5AT competed with T13Ha on their previous substrate taxadien-5α-ol. Therefore, this gene is important, because it is located at the beginning of the pathway and on the branch. This is a very low-expression gene that requires the use of a high level of elicitors to identify it. Herein, for the first time, the full length and features of the *TDAT* gene was identified through bioinformatics analysis instead of through costly, time–consuming, and labor-intensive work. Understanding the sequence of *TDAT* will be important to PC engineering in future studies.

Author contribution statement

The project was designed and Supervised by SAR. MRS performed all the experiments, analyzed data, and co-wrote the paper. SRM helped to identify the gene and co-supervised the research. JZ helped to analyze the data. AL helped to analyze the tertiary structure of the protein. All authors discussed the results and contributed to the final manuscript.

Funding information

This research did not receive any specific grant from funding agencies in the public, commercial, or not-for-profit sectors.

Competing of interest

The authors declare that they have no competing interests.

Data availability statement

The datasets generated and/or analyzed during the current study are included in the article and are available from the corresponding author on reasonable request.

References

1. Pandi M, SenthilKumaran R, Rajapriya P, Yogeswari S, Muthumary J. Taxol, A potential drug for the treatment of cancer. Biores Bull. 2013;2(1):1-9.
2. Agarwal G, Carcache PJB, Addo EM, Kinghorn AD. Current status and contemporary approaches to the discovery of antitumor agents from higher plants. Biotechnology Advances. 2020;38. doi: https://doi.org/10.1016/j.biotechadv.2019.01.004.
3. Strobel GA, Hess WM, Ford E, Sidhu R, Yang X. Taxol from fungal endophytes and the issue of biodiversity. Journal of Industrial Microbiology & Biotechnology. 1996;17(5):417-23.
4. Expósito O, Bonfill M, Moyano E, Onrubia M, Mirjalili M, Cusido R, et al. Biotechnological production of taxol and related taxoids: current state and prospects. Anti-Cancer Agents in Medicinal Chemistry (Formerly Current Medicinal Chemistry-Anti-Cancer Agents). 2009;9(1):109-21.
5. Stanton RA, Gernert KM, Nettles JH, Aneja R. Drugs that target dynamic microtubules: a new molecular perspective. Medicinal research reviews. 2011;31(3):443-81.
6. Hoffman A, Shahidi F. Paclitaxel and other taxanes in hazelnut. Journal of Functional Foods. 2009;1(1):33-7. doi: https://doi.org/10.1016/j.jff.2008.09.004.
7. Rahimi N, Nazeri S, Mirzaei S. Taxol production by endophytic fungi isolated from *Corylus avellana*. Arak Medical University Journal. 2013;16(72):37-44.
8. Vasundhara M, Reddy MS, Kumar A. Chapter 18 - Secondary Metabolites From Endophytic Fungi and Their Biological Activities. In: Gupta VK, Pandey A, editors. New and Future Developments in Microbial Biotechnology and Bioengineering. Amsterdam: Elsevier; 2019. p. 237-58.
9. Heinig U, Scholz S, Jennewein S. Getting to the bottom of Taxol biosynthesis by fungi. Fungal diversity. 2013;60(1):161-70.
10. Gangadevi V, Muthumary J. A novel endophytic taxol-producing fungus Chaetomella raphigera isolated from a medicinal plant, *Terminalia arjuna*. Applied biochemistry and biotechnology. 2009;158(3):675-84.
11. Malik S, Cusidó RM, Mirjalili MH, Moyano E, Palazón J, Bonfill M. Production of the anticancer drug taxol in *Taxus baccata* suspension cultures: a review. Process Biochemistry. 2011;46(1):23-34.
12. Hefner J, Ketchum RE, Croteau R. Cloning and Functional Expression of a cDNA Encoding Geranylgeranyl Diphosphate Synthase fromTaxus canadensisand Assessment of the Role of this Prenyltransferase in Cells Induced for Taxol Production. Archives of biochemistry and biophysics. 1998;360(1):62-74.
13. Abbasin Z, Zamani S, Movahedi S, Khaksar G, Tabatabaei BS. In vitro micropropagation of Yew (Taxus baccata) and production of plantlets. Biotechnology. 2010;9(1):48-54.
14. Bestoso F, Ottaggio L, Armirotti A, Balbi A, Damonte G, Degan P, et al. In vitro cell cultures obtained from different explants of Corylus avellana produce Taxol and taxanes. BMC biotechnology. 2006;6(1):45.
15. Ottaggio L, Bestoso F, Armirotti A, Balbi A, Damonte G, Mazzei M, et al. Taxanes from shells and leaves of Corylus avellana. Journal of natural products. 2007;71(1):58-60.
16. Stephanopoulos G, Aristidou AA, Nielsen J. Metabolic engineering: principles and methodologies. San Diego, California: Academic Press, Elsevier; 1998.
17. Kai G, Miao Z, Qiu C, Zhang L, Zhao L, Li Z, et al. Molecular cloning and characterization of a taxadienol acetyl transferase cDNA from Taxus x media. Plant science. 2004;167(4):759-64.
18. Wang Y, Miao Z, Tang K. Molecular cloning and functional expression analysis of a new gene encoding geranylgeranyl diphosphate synthase from hazel (Corylus avellana L. Gasaway). Molecular biology reports. 2010;37(7):3439-44.
19. Walker K, Croteau R. Taxol biosynthetic genes. Phytochemistry. 2001;58(1):1-7.
20. Wang Y, Guo B, Zhang F, Yao H, Miao Z, Tang K. Molecular cloning and functional analysis of the gene encoding 3-hydroxy-3-methylglutaryl coenzyme A reductase from hazel (Corylus avellana L. Gasaway). BMB Reports. 2007;40(6):861-9.
21. Maheshwari P, Garg S, Kumar A. Taxoids: Biosynthesis and in vitro production. Biotechnology and Molecular Biology Reviews. 2008;3(4):71-87.
22. Croteau R, Ketchum RE, Long RM, Kaspera R, Wildung MR. Taxol biosynthesis and molecular genetics. Phytochemistry Reviews. 2006;5(1):75-97.
23. Walker K, Croteau R. Taxol biosynthesis: a review of some determinant steps. Recent Advances in Phytochemistry (Netherlands). 1999.
24. Nims E, Dubois CP, Roberts SC, Walker EL. Expression profiling of genes involved in paclitaxel biosynthesis for targeted metabolic engineering. Metabolic engineering. 2006;8(5):385-94.
25. Raeispour shirazi M, Rahpeyma SA, Zolala J. A new approach to prevent callus browning by modification of sub-culture method in hazelnut (*Corylus avellana* L.). Biologia Plantarum. 2020;64:417-21. doi: DOI: 10.32615/bp.2020.009.

26. Rahpeyma SA, Moieni A, Jalali Javaran M. Paclitaxel production is enhanced in suspension-cultured hazel (*Corylus avellana* L.) cells by using a combination of sugar, precursor, and elicitor. Engineering in Life Sciences. 2015;15(2):234-42.
27. Tamura K, Stecher G, Peterson D, Filipski A, Kumar S. MEGA6: molecular evolutionary genetics analysis version 6.0. Molecular biology and evolution. 2013;30(12):2725-9.
28. Gao F, Chen C, Arab DA, Du Z, He Y, Ho SYW. EasyCodeML: A visual tool for analysis of selection using CodeML. Ecol Evol. 2019;9(7):3891-8. doi: 10.1002/ece3.5015. PubMed PMID: 31015974.
29. Nei M, Gojobori T. Simple methods for estimating the numbers of synonymous and nonsynonymous nucleotide substitutions. Molecular biology and evolution. 1986;3(5):418-26.
30. Yang Z, Wong WS, Nielsen R. Bayes empirical Bayes inference of amino acid sites under positive selection. Molecular biology and evolution. 2005;22(4):1107-18.
31. Walker JM. The proteomics protocols handbook: Springer; 2005.
32. Madhusudhan M, Marti-Renom MA, Eswar N, John B, Pieper U, Karchin R, et al. Comparative protein structure modeling. The Proteomics Protocols Handbook: Springer; 2005. p. 831-60.
33. Morris AL, MacArthur MW, Hutchinson EG, Thornton JM. Stereochemical quality of protein structure coordinates. Proteins: Structure, Function, and Bioinformatics. 1992;12(4):345-64.
34. Lallemand LA, Zubieta C, Lee SG, Wang Y, Acajjaoui S, Timmins J, et al. A structural basis for the biosynthesis of the major chlorogenic acids found in coffee. Plant physiology. 2012:pp. 112.202051.
35. Rosin CD, Belew RK, Morris GM, Olson AJ, Goodsell DS. Coevolutionary analysis of resistance-evading peptidomimetic inhibitors of HIV-1 protease. Proceedings of the National Academy of Sciences. 1999;96(4):1369-74.
36. Yang Q, Reinhard K, Schiltz E, Matern U. Characterization and heterologous expression of hydroxycinnamoyl/benzoyl-CoA: anthranilate N-hydroxycinnamoyl/benzoyltransferase from elicited cell cultures of carnation, Dianthus caryophyllus L. Plant molecular biology. 1997;35(6):777-89.
37. Gallego A, Malik S, Yousefzadi M, Makhzoum A, Tremouillaux-Guiller J, Bonfill M. Taxol from Corylus avellana: paving the way for a new source of this anti-cancer drug. Plant Cell, Tissue and Organ Culture (PCTOC). 2017:1-16.
38. Walker K, Long R, Croteau R. The final acylation step in taxol biosynthesis: cloning of the taxoid C13-side-chain N-benzoyltransferase from Taxus. Proceedings of the National Academy of Sciences. 2002;99(14):9166-71.
39. Tu J, Zhu P, Cheng K-d, Liu J. Cloning and expression of Taxus acyltransferase cDNA. Zeitschrift für Naturforschung C. 2004;59(9-10):755-61.
40. Lallemand LA, McCarthy JG, McSweeney S, McCarthy AA. Purification, crystallization and preliminary X-ray diffraction analysis of a hydroxycinnamoyl-CoA shikimate/quinate hydroxycinnamoyltransferase (HCT) from Coffea canephora involved in chlorogenic acid biosynthesis. Acta Crystallographica Section F: Structural Biology and Crystallization Communications. 2012;68(7):824-8.

Supplementary captions

Supplementary figure S1. Agarose (1%) gel electrophoresis of nested-PCR analysis of *C. avellana*; Ladder (L) 100bp,1: Forward 1 + Reverse 1 (Fr_1+Rr_1), 2. Forward 1+ Reverse 2 (Fr_1+Rr_2), 3. Forward 2 + Reverse 1 (Fr_2+Rr_1), 4. Forward 2 + Reverse 2 (Fr_2+Rr_2), 5. Positive control (GAPDH primers), 6. Negative control (water).

Supplementary table S1. The results of ORFs' online software; the most portable framework and the tallest of ORF are in + 1 and the nucleotide of 97 to 1398.

Supplementary figure S2 Evolutionary relationships of *C. avellana* L. *TDAT* gene with 15 geneses, based on the branch-site model, by using the EasyCodeML software (PAML package).

Supplementary figure S3. The Ramachandran plot and the frequency of amino acids in a different position for *C. avellana* L. (A) 4G0B template (B) MO323. In the picture, [A, B, L] show a very favored region for amino acids. [a, b, l, p] show the allowed zone, and [~a, ~b, ~l, ~p] show the permitted regions with ignorance.

 Click here to access/download;Figure;fig 1.tif

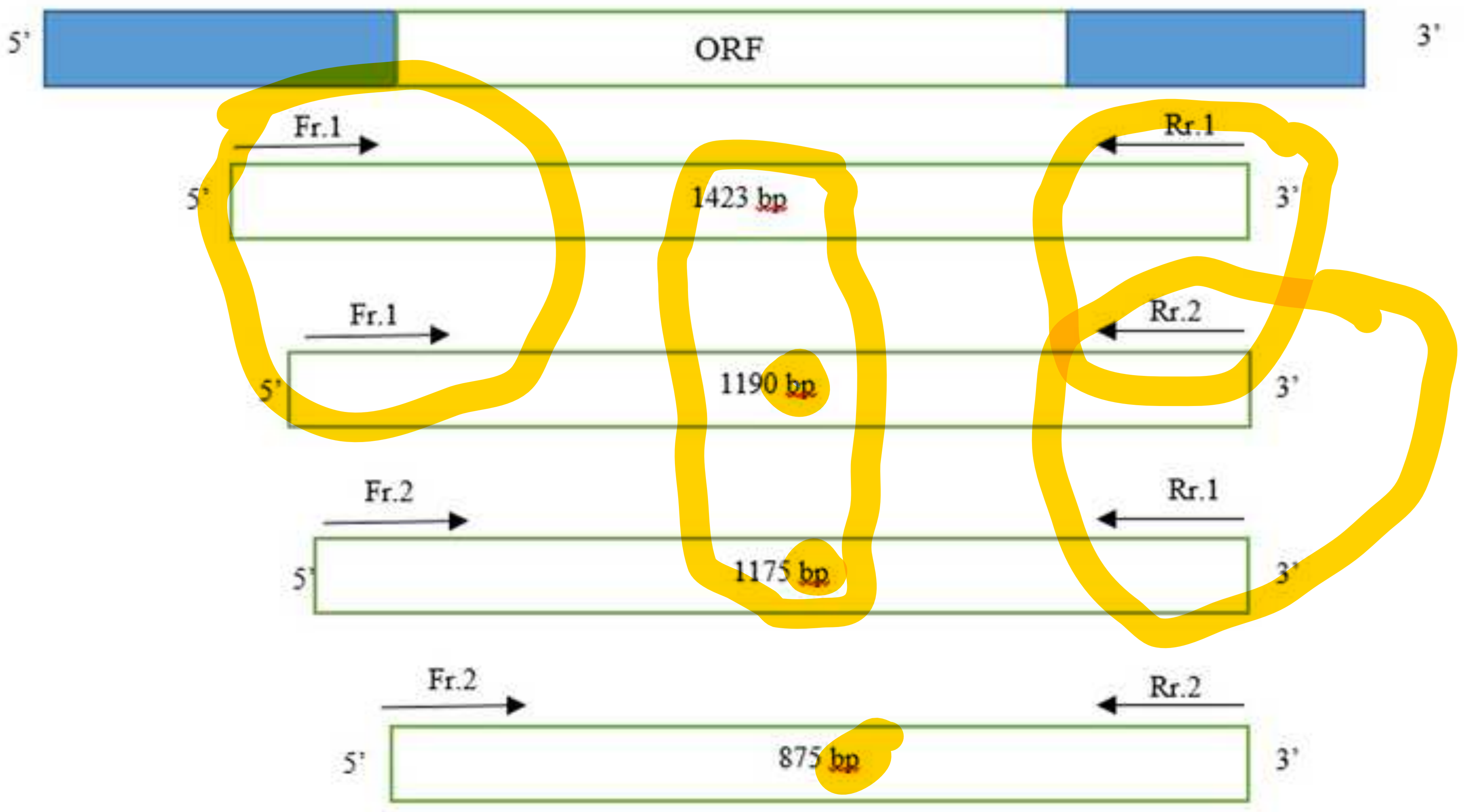

Click here to access/download;Figure;fig 2a .tif

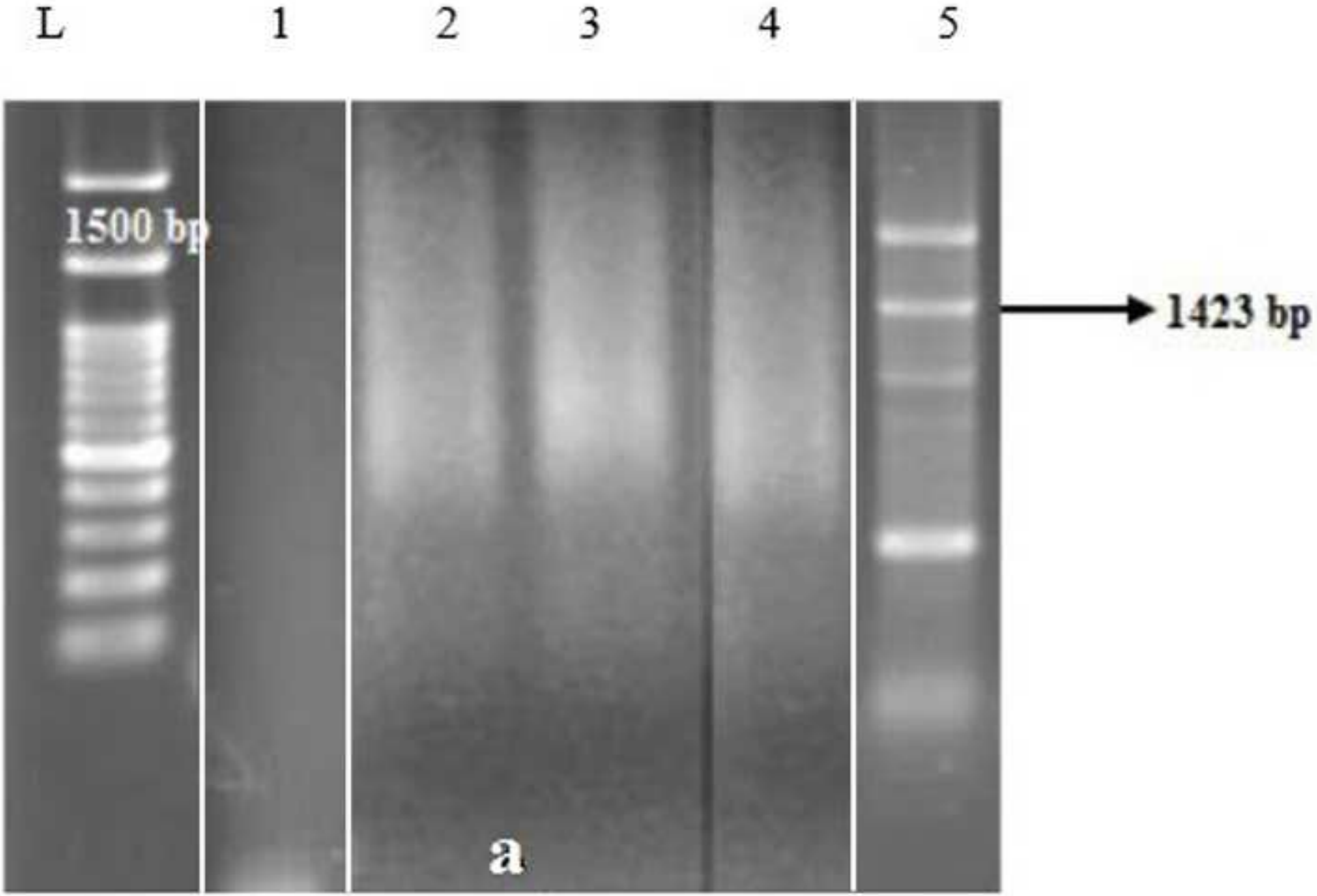

Click here to access/download;Figure;fig 2b .tif

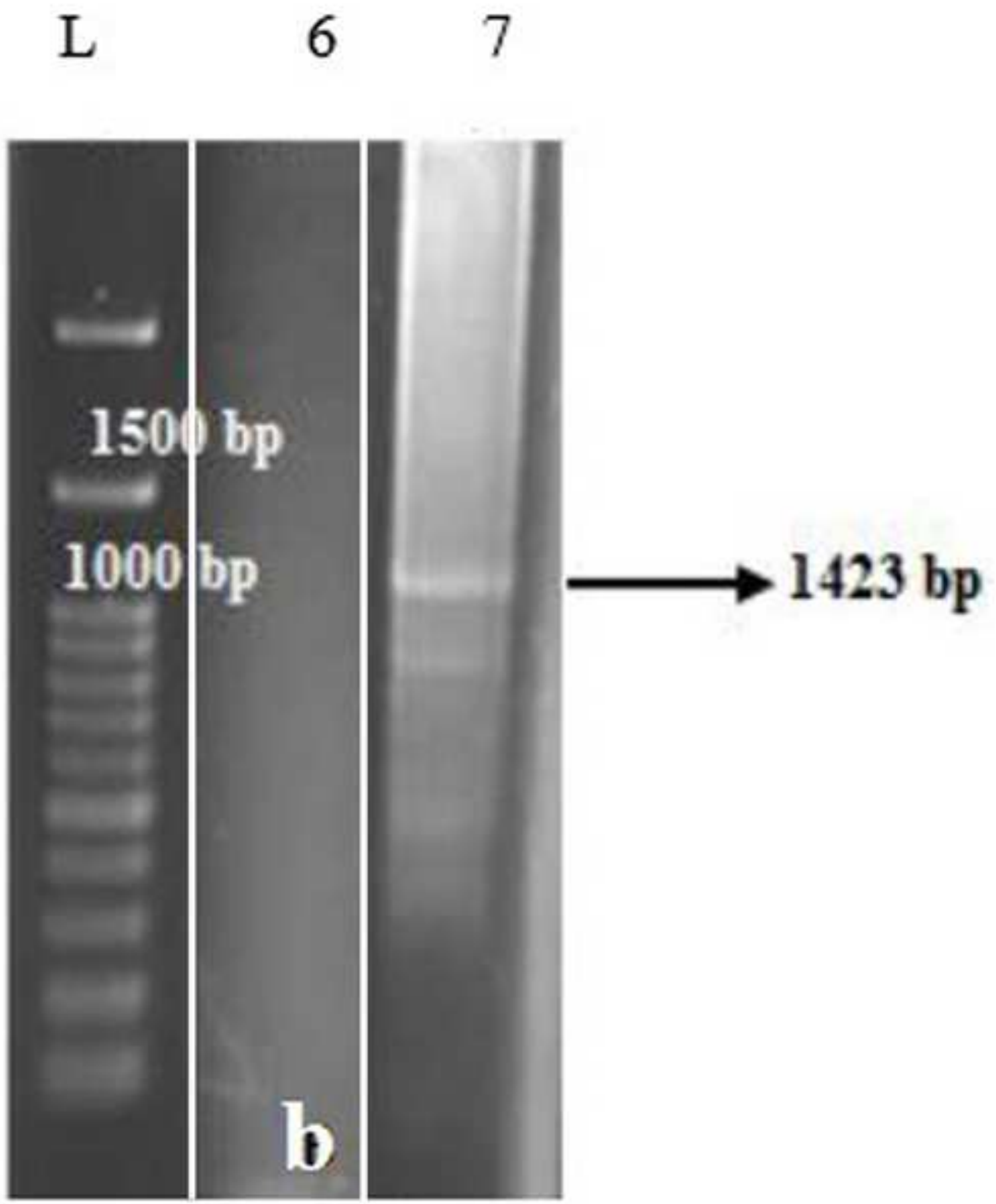

Figure 3

Click here to access/download;Figure;Fig 3.tif

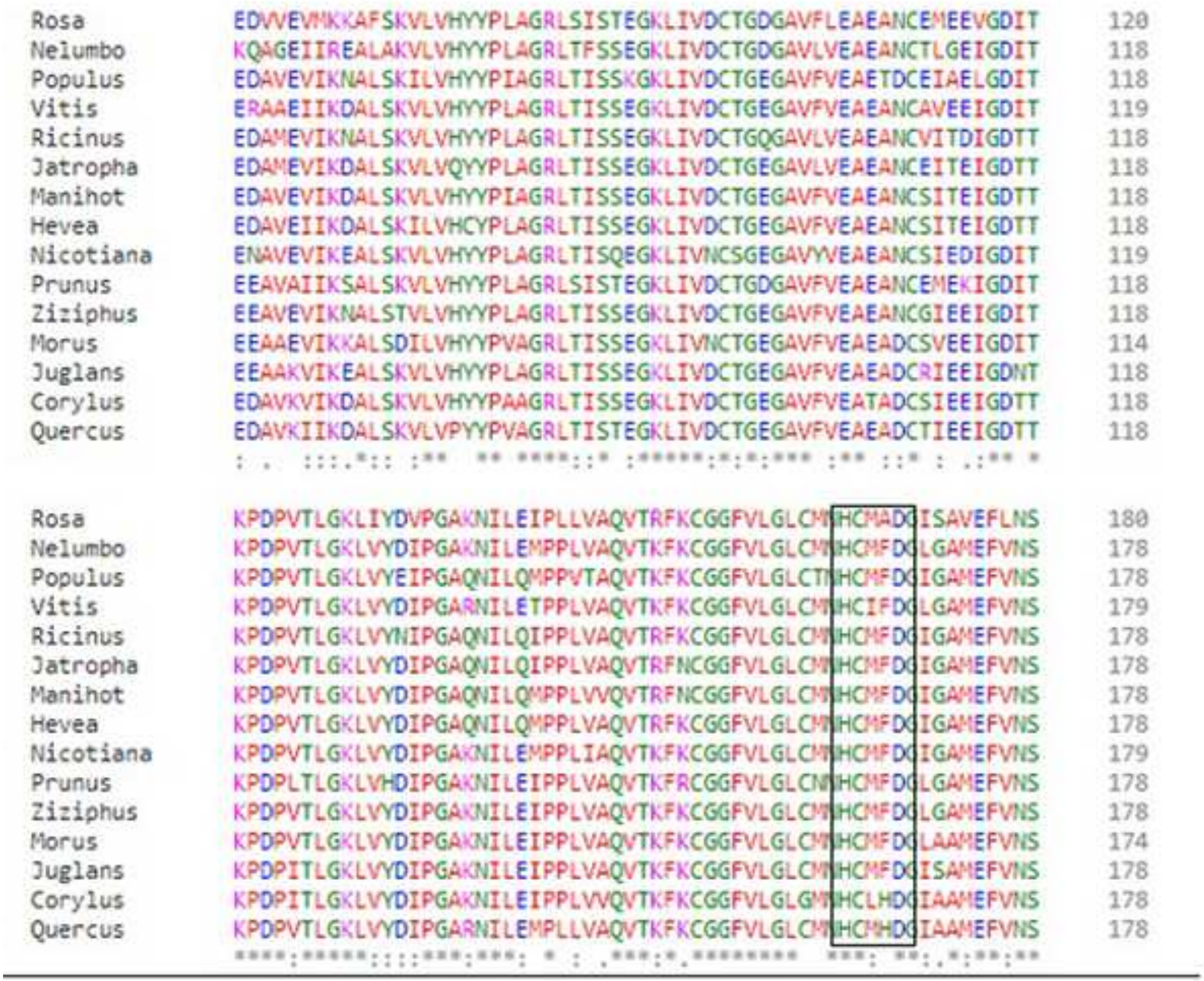

Figure 4

Click here to access/download;Figure;fig 4.tif

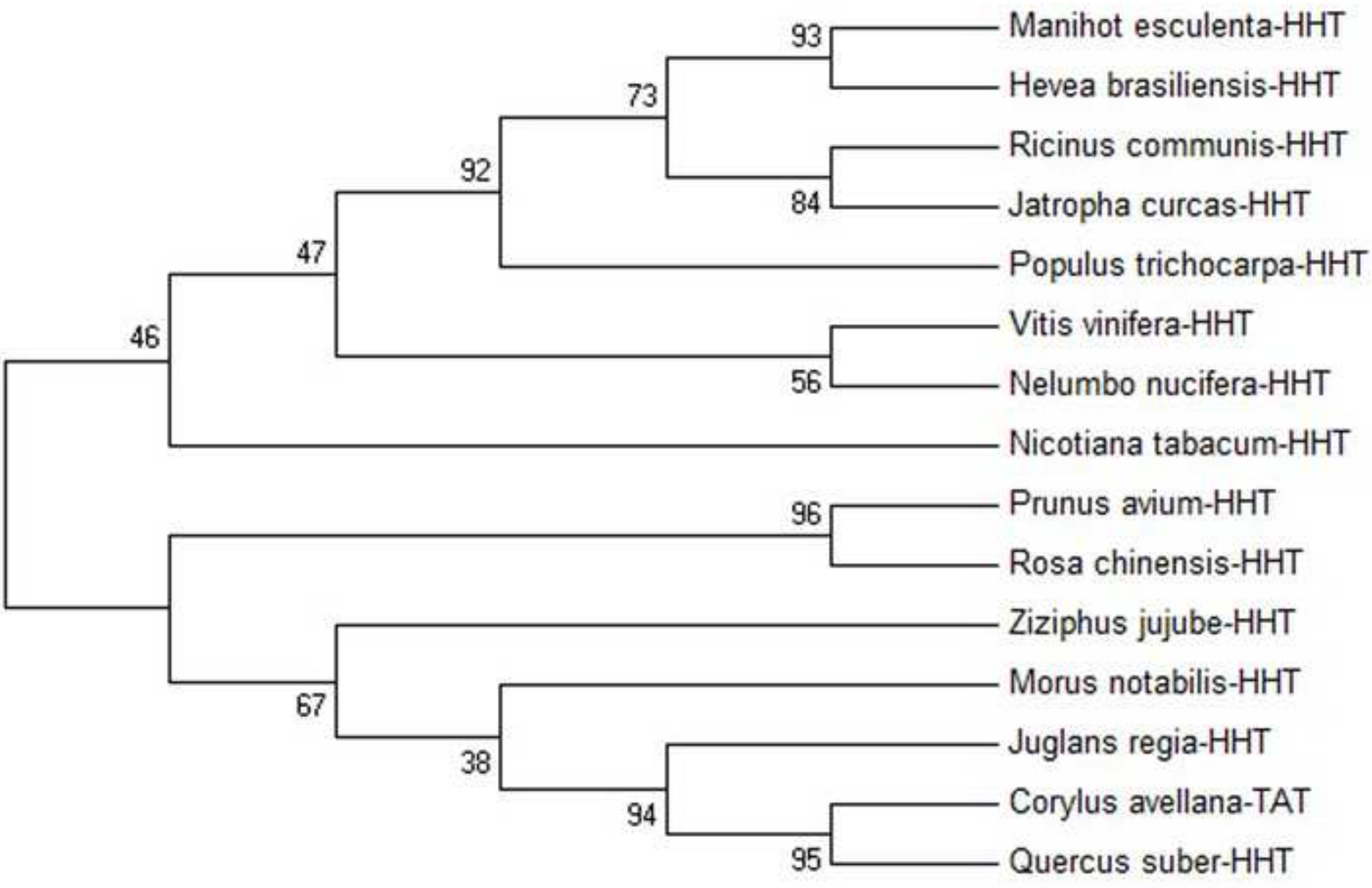

Figure 5

Click here to access/download;Figure;fig 5.tif

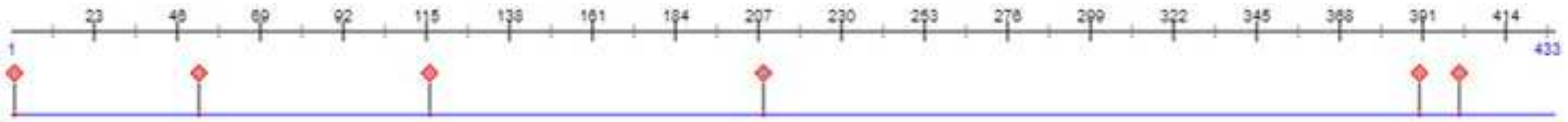

Figure 6

Click here to access/download;Figure;fig 6 (L&R).tif

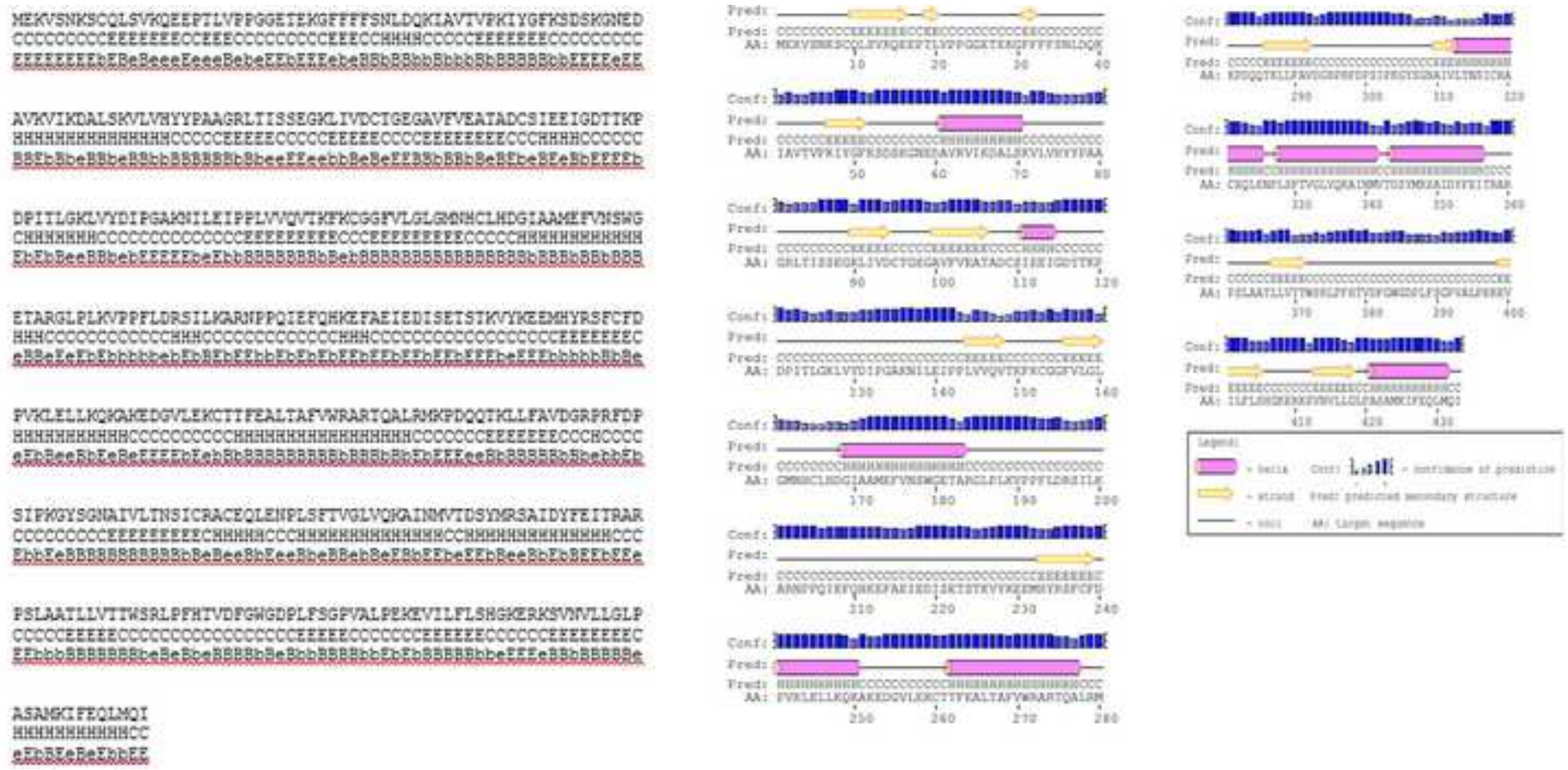

Figure 7

Click here to access/download;Figure;fig 7.tif

```
  1 MEKVSNKSCQLSVKQEEPTLVPPGGETEKGFFFFSNLDQKIAVTVPKIYGFKSDSKGNED 60
                  ++++++++++++++

 61 AVKVIKDALSKVLVHYYPAAGRLTISSEGKLIVDCTGEGAVFVEATADCSIEEIGDTTKP 120


121 DPITLGKLVYDIPGAKNILEIPPLVVQVTKFKCGGFVLGLGMNHCLHDGIAAMEFVNSWG 180
                    OOOOOOOOOOOOO

181 ETARGLPLKVPPFLDRSILKARNPPQIEFQHKEFAEIEDISETSTKVYKEEMHYRSFCFD 240


241 PVKLELLKQKAKEDGVLEKCTTFEALTAFVWRARTQALRMKPDQQTKLLFAVDGRPRFDP 300


301 SIPKGYSGNAIVLTNSICRACEQLENPLSFTVGLVQKAINMVTDSYMRSAIDYFEITRAR 360
                       OOOOOOOOOOOOOOOOO

361 PSLAATLLVTTWSRLPFHTVDFGWGDPLFSGPVALPEKEVILFLSHGKERKSVNVLLGLP 420
                      OOOOOOOOOOOOOOOOOOO

421 ASAMKIFEQLMQI 433


    Symbols   PEST motifs
    +++++++   potential
    OOOOOOO   poor
```

Figure 8

Click here to access/download;Figure;fig 8.tif

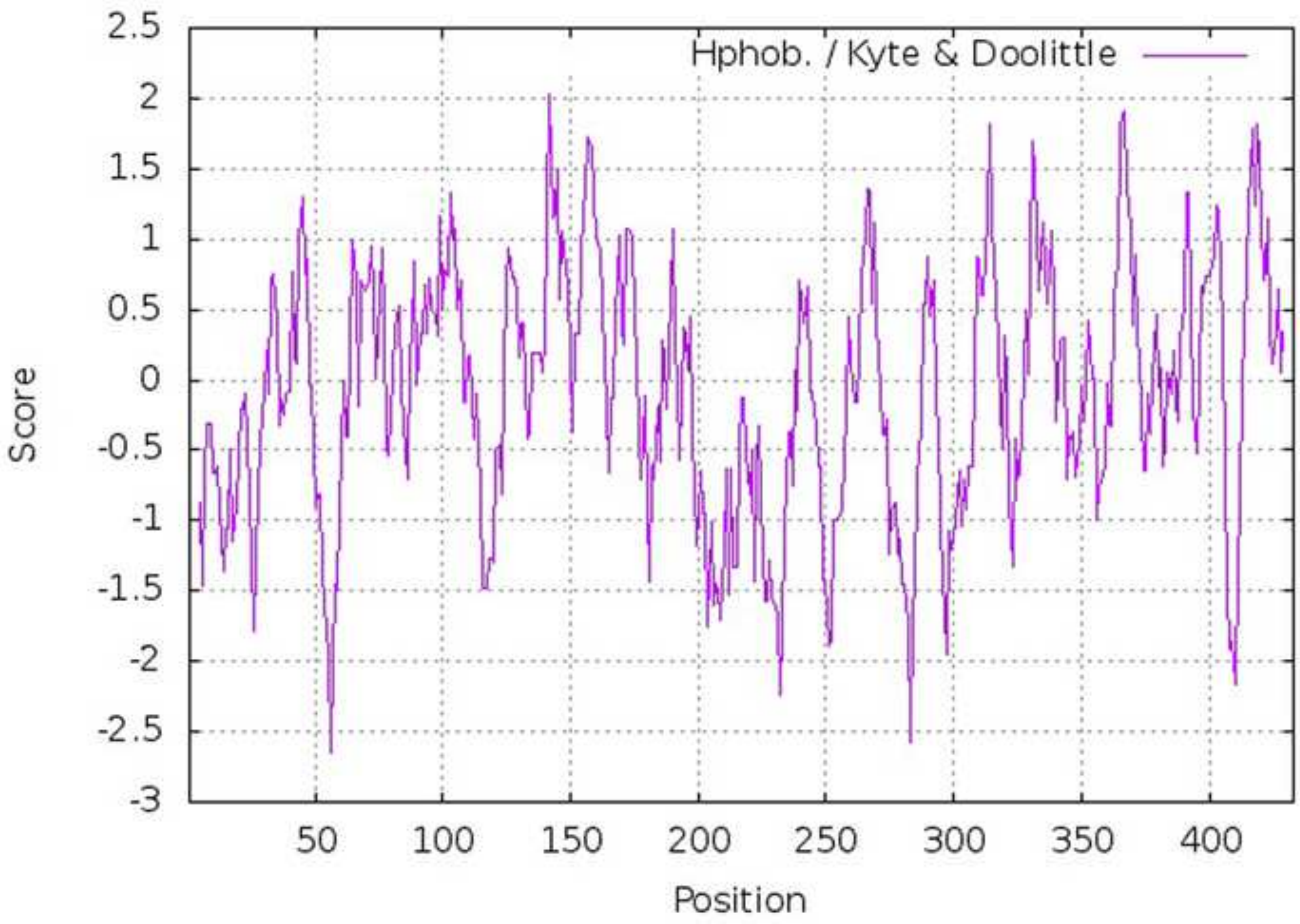

Figure 9A Click here to access/download;Figure;fig 9a .tif

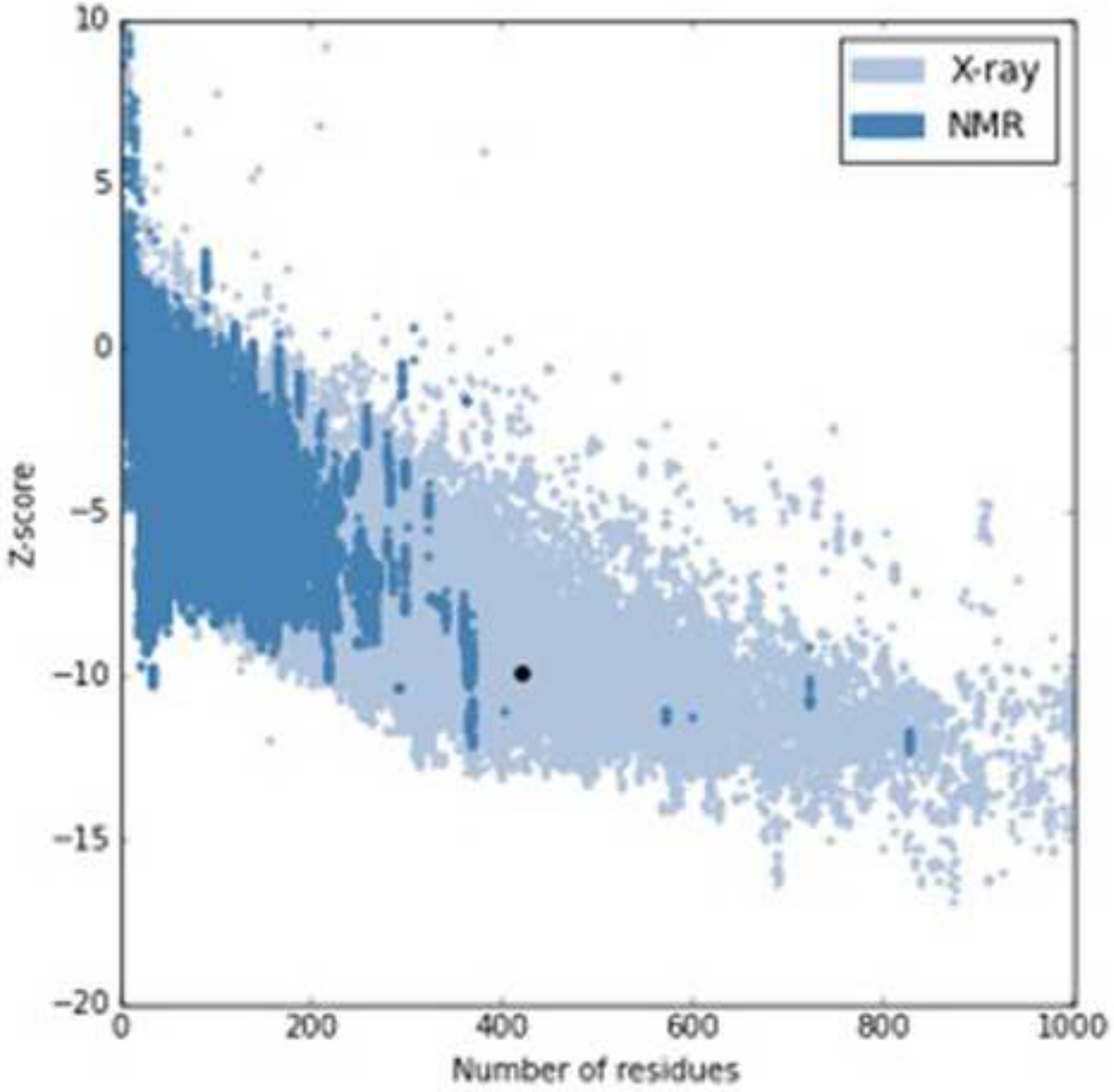

a

 Click here to access/download;Figure;fig 9b .tif

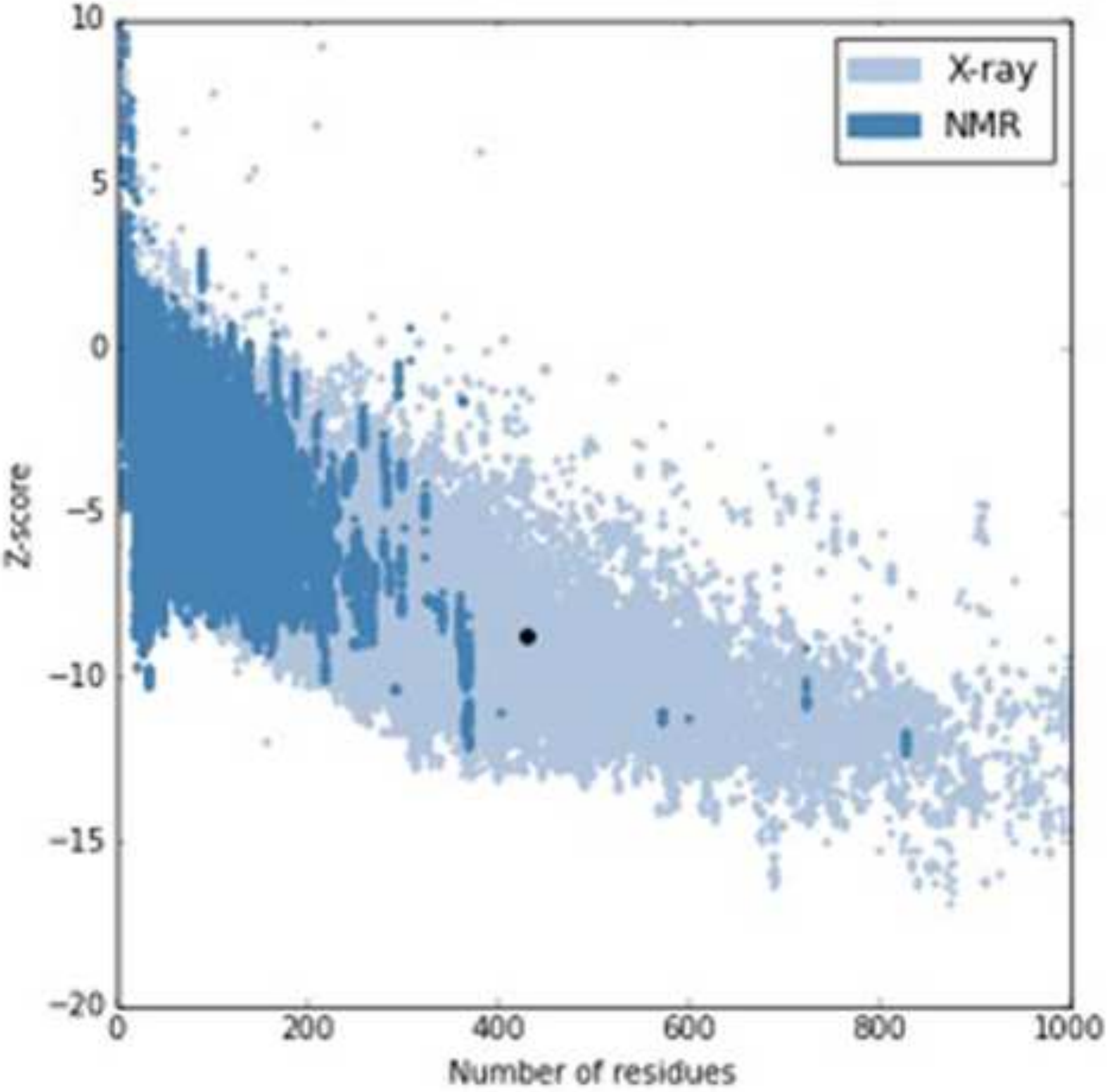

b

Figure 10

Click here to access/download;Figure;fig 10.tif

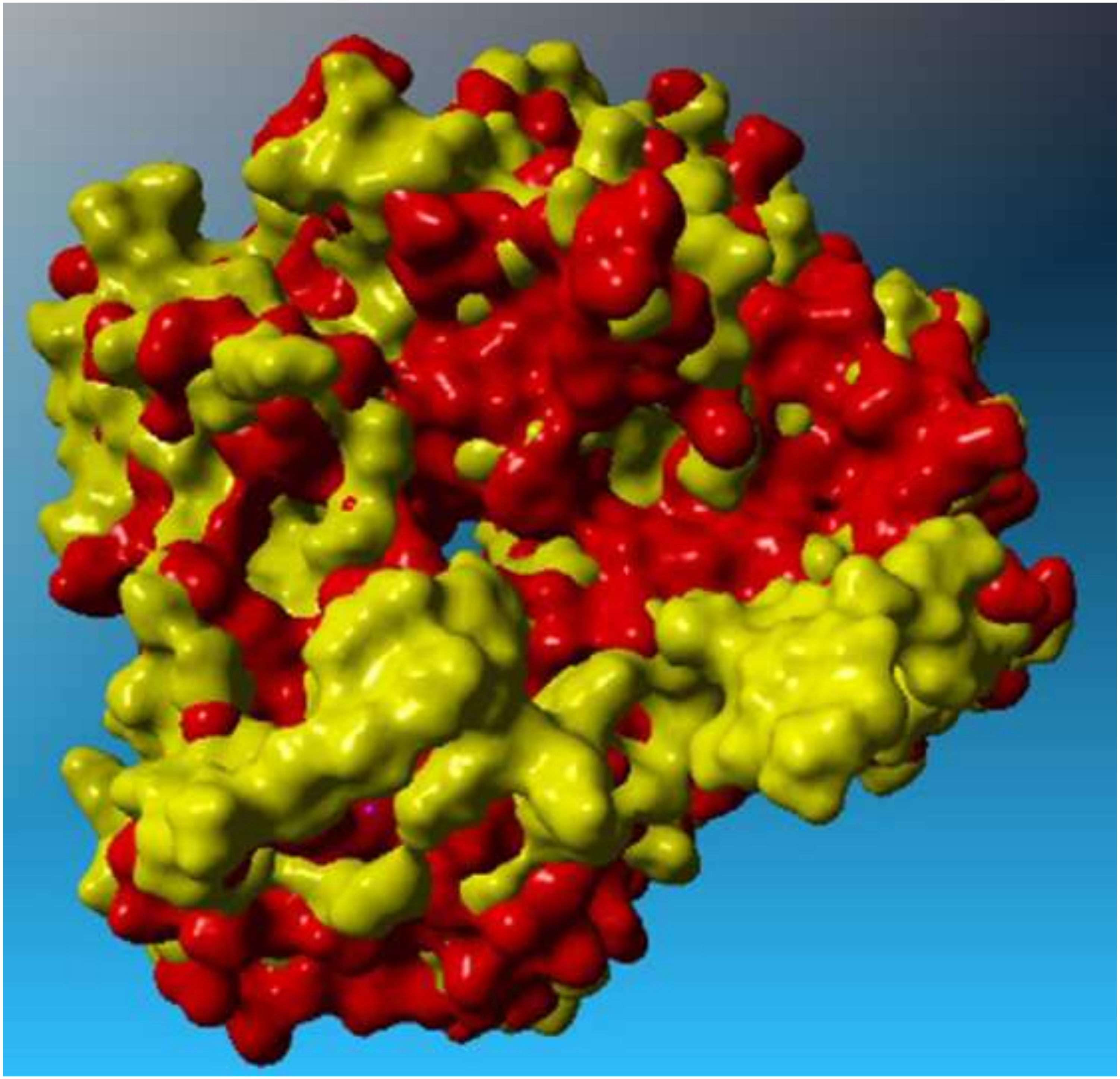

Figure 11A

Click here to access/download;Figure;fig 11 a.tif

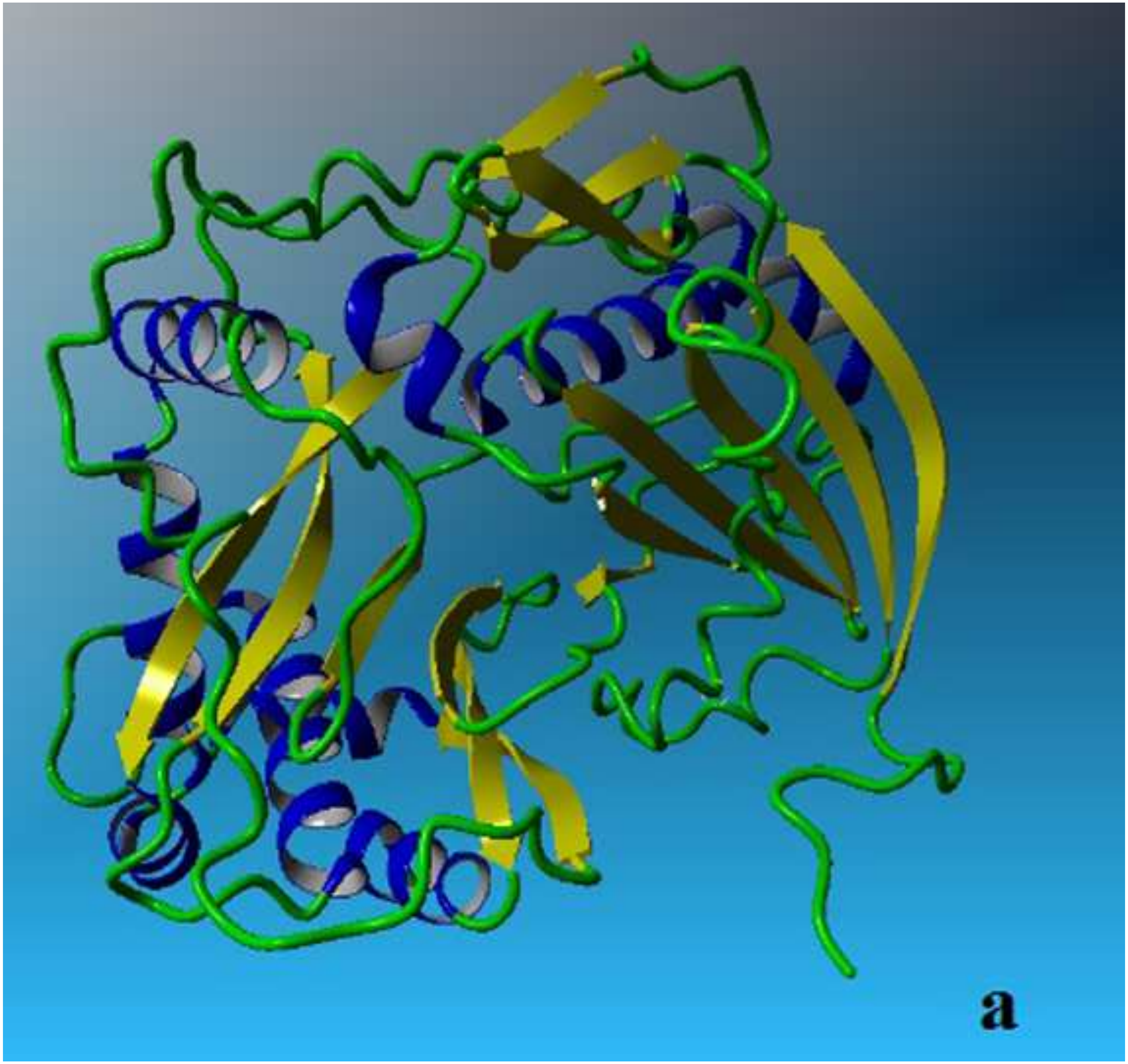

Figure 11B

Click here to access/download;Figure;fig 11 b.tif

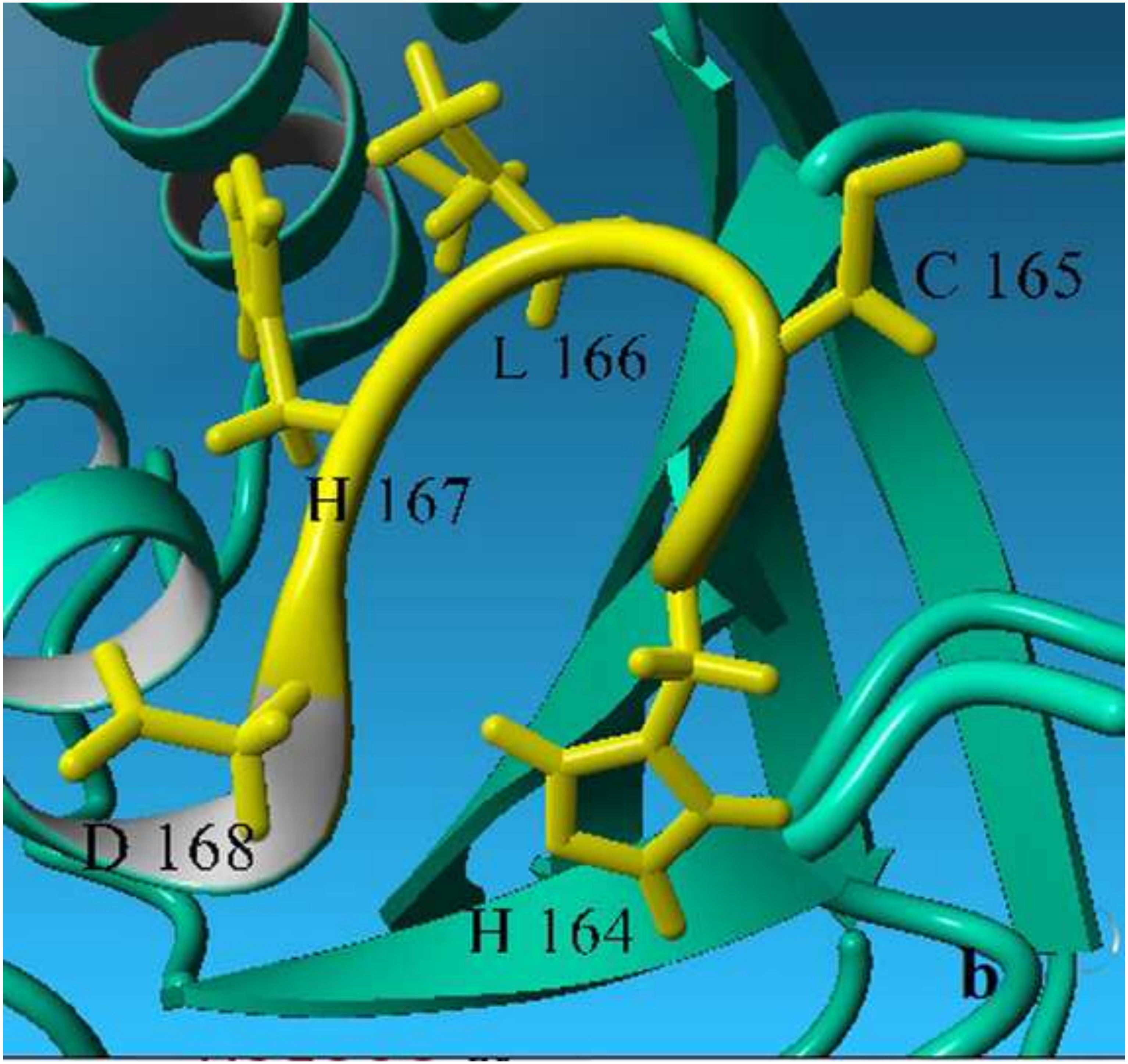

Figure S1

Click here to access/download;Figure;fig S1.tif

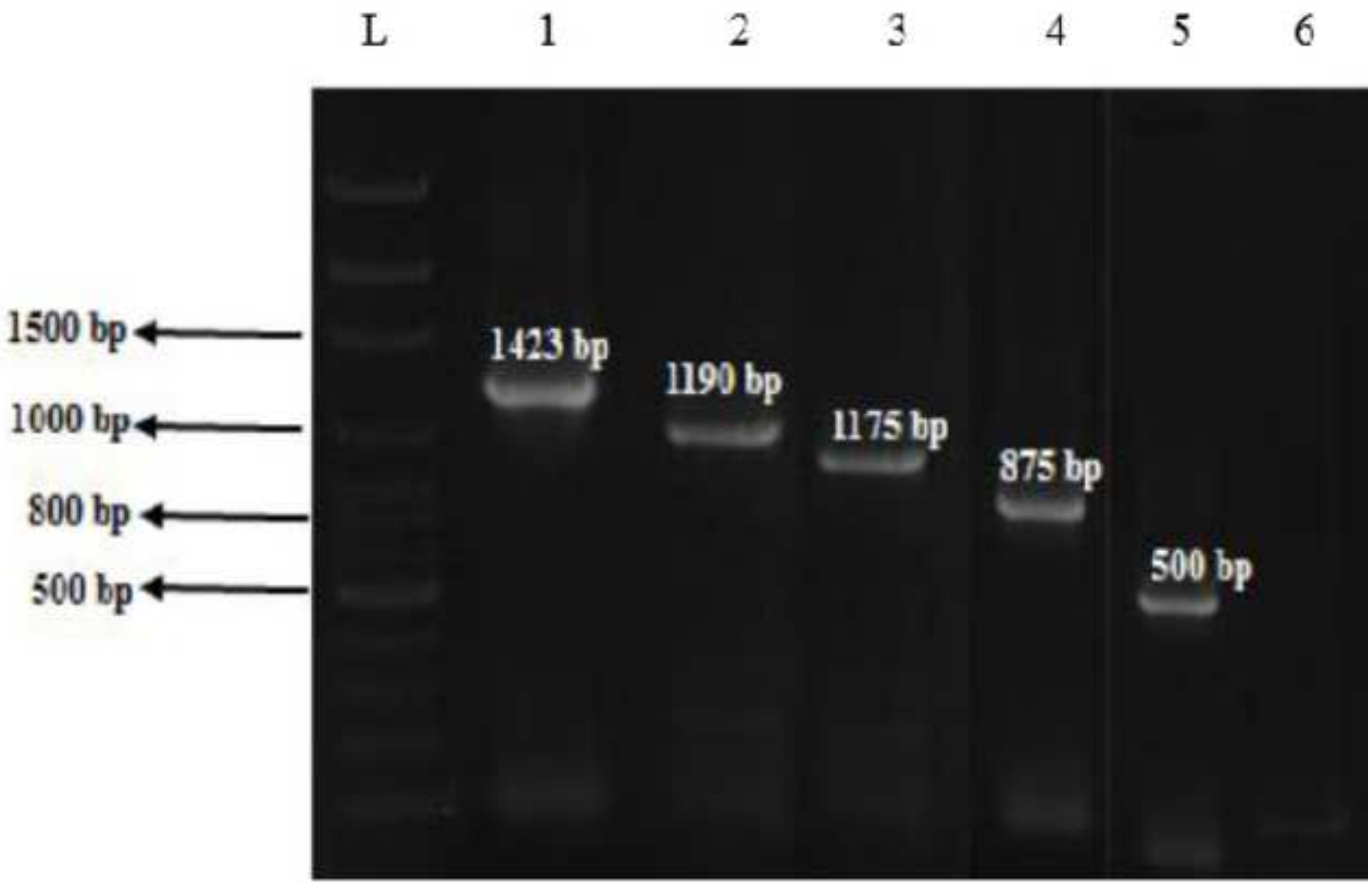

Figure S2

Click here to access/download;Figure;fig S2.tif

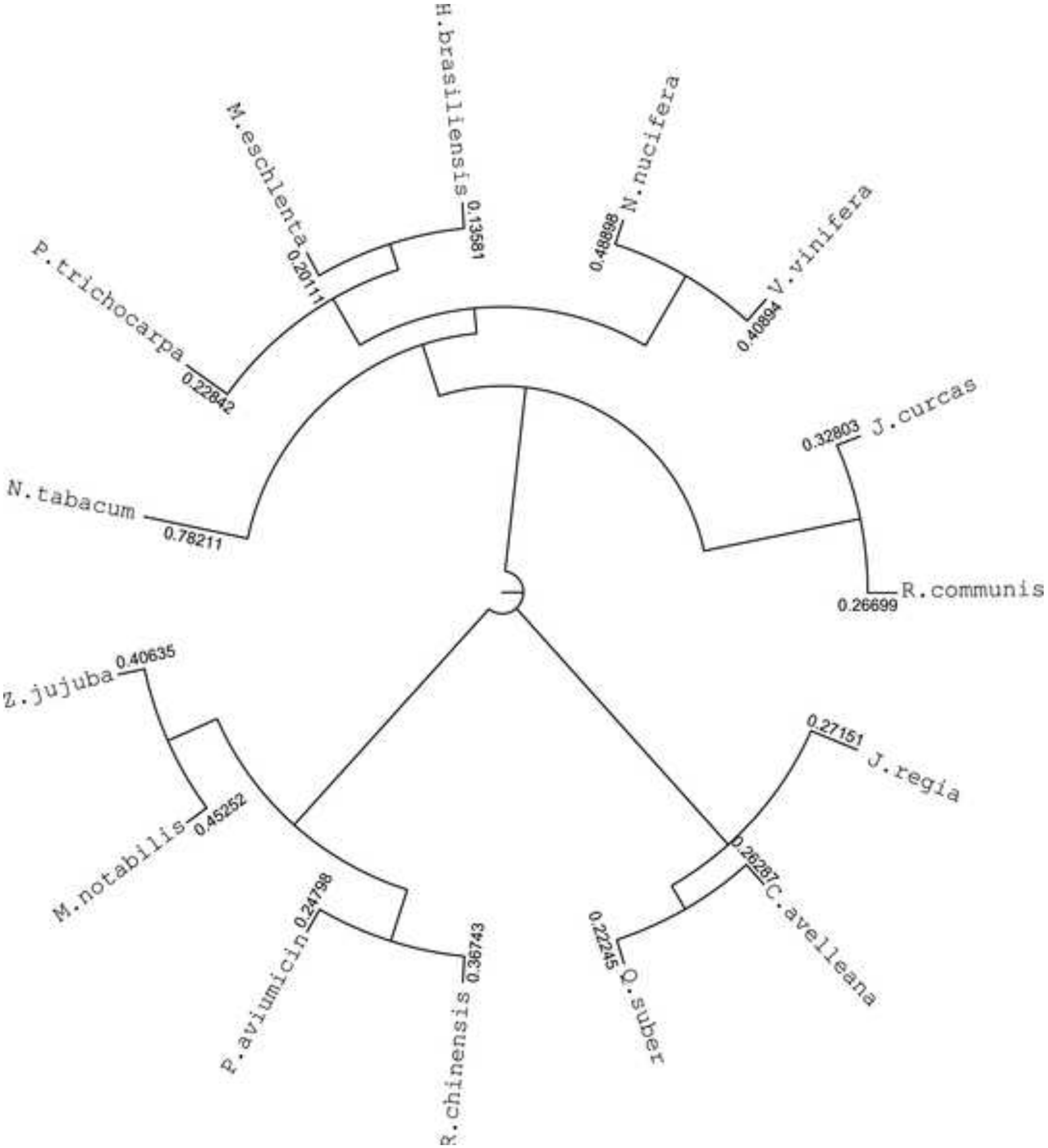

Figure S3

Click here to access/download;Figure;fig S3.tif

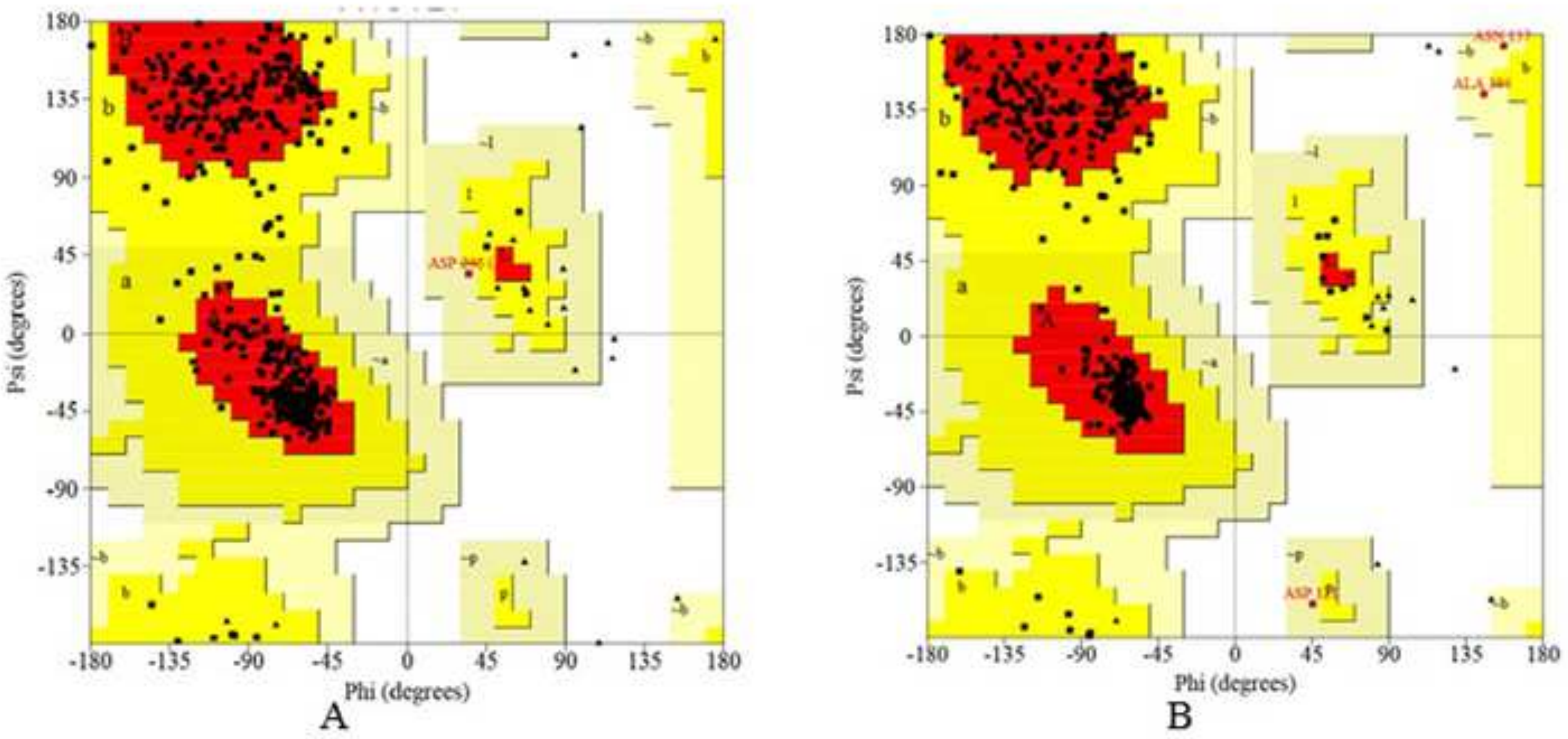

Click here to access/download
Supporting Information
Supplementary figures and table.docx
