# Supplementary material for: Identification and in-silico characterization of taxadien-5α-ol-O-acetyltransferase (TDAT) gene in Corylus avellana L
Source: PLoS One. 2021 Aug 27;16(8):e0256704. doi: 10.1371/journal.pone.0256704 (PMC8396717; doi:10.1371/journal.pone.0256704)
Supplement: Attachment — Submitted filename: responses to reviewer.docx [file pone.0256704.s012.docx]

Dear Editor-in-Chief,

We would like to deeply thank the editorial office and the reviewers for the constructive comments which helped us to improve the paper and thank you for giving us the opportunity to submit a revised draft of our manuscript titled "Identification and *in-silico* Characterization of *taxadien-5α-ol-O-acetyltransferase (TDAT*) Gene in *Corylus avellana* L". The authors tried to address all reviewers’ comments in the revised manuscript. We hope our revision has improved the paper to a level of their satisfaction. Number-wise Responses to the specific editorial comments, suggestions, and queries are given below; the revisions in the manuscript have been marked highlighted with track changes in a file labeled ‘ Revised manuscript with track changes.

Enclosed, please find the revised version of the manuscript entitled:

“Identification and *in-silico* Characterization of *taxadien-5α-ol-O-acetyltransferase (TDAT*) Gene in *Corylus avellana* L.

By:

Mona Raeispour Shirazi, Sara Alsadat Rahpeyma, Sajad Rashidi Monfared, Jafar Zolala and Azadeh Lohrasbi-Nejad^1^

Sincerely yours,

Dr. Sara Alsadat Rahpeyma

Assistant Professor of Plant Breeding

Department of Agricultural Biotechnology

Faculty of Agriculture

Shahid Bahonar University of Kerman

Iran

**Responses to journal requirements:**

1. Please ensure that your manuscript meets PLOS ONE's style requirements, including those for file naming.

- The authors tried to improve and adjust the manuscript style to meet PLOS ONE’s style found at <https://journals.plos.org/plosone/s/file?id=wjVg/PLOSOne_formatting_sample_main_body.pdf> and [https://journals.plos.org/plosone/s/file?id=ba62/PLOSOne_formatting_sample_title_authors_affiliations.pdf](javascript:false). Track changes were used to mark the changes, including text format, figures, tables, references, grammatical adjustments, etc, across the updated manuscript.

2- PLOS ONE now requires that authors provide the original uncropped and unadjusted images underlying all blot or gel results reported in a submission’s figures or Supporting Information files.

- Two gel figures are included in the manuscript "PONE-D-21-05459" (Fig 2. and S1 Fig). The authors attempted to compile original gel figs in a single PDF file format called "S1-row pictures," which contains all the original and unadjusted gel images, included in the manuscript’s main figures and supplemental figures. **Fig 2.** is in the public data presented in the main article and **S1 Fig** is in the ‘supporting information’ section. Also, the author tried to consider all requirements for gel reporting, such as labeling and annotating the experimental and not included lanes, molecular markers’ weight, and so on. Unfortunately, we lost all of the original gel images due to a serious problem with my student (Mona Raeispour) laptop, and we only have cropped images retrieved from emails. But I, as the corresponding author, ensure the editorial board that no manipulating has been done in figures. The cropped lanes of images were identified with the blanks among the parts. The photos were captured using the VIBER Company's Quantum gel documentation imaging technology. The authors hope that the revision will meet PLOS ONE's approval.

Response to Comments by Reviewer 1

We'd like to express our gratitude to the Reviewer for his or her constructive comments and suggestions. Based on his/her remarks, various revisions were made to improve the quality of the manuscript. The modifications were highlighted with track changes through the revised manuscript. The next sections contain our responses to the dear reviewer comments

**Comment No.1**: This manuscript presents interesting bioinformatic findings about TAT in hazelnuts species, which will be interesting to the community, specially the prediction of secondary and tertiary structures. Nevertheless it needs some serious improvements in the literature review as well as how the data and figures are explained and presented.

**Response:** Thanks to the reviewer for the kind comment on the manuscript. The author tried to improve the literature review by including more recent studies and providing additional details about the presented study. The modifications have been addressed in the text as well as in the separate responses to the attached comments.

**Comment No.2**: Some figures are blurry and couldn’t be assessed properly.

**Response:** We considered the reviewer’s suggestion and Figs 1, 2, 3, 6, and 9 changed to more appropriate ones. All figures were checked to meet journal style.

**Comment No.3**: It needs to compare more with recents studies where the TAT has been utilised and highlight more the relevance of the work too.

**Response:** To highlight the significance of the subject, the recent and relevant TAT studies were added to the text throughout the introduction and discussion sections. They are addressed via track changes.

The following new references were added to the introduction and discussion:

- Salehi M, Moieni A, Safaie N, Farhadi S. Whole fungal elicitors boost paclitaxel biosynthesis induction in Corylus avellana cell culture. Plos one. 2020;15(7):e0236191.
- Yu C, Zhang C, Xu X, Huang J, Chen Y, Luo X, et al. Omic analysis of the endangered Taxaceae species Pseudotaxus chienii revealed the differences in taxol biosynthesis pathway between Pseudotaxus and Taxus yunnanensis trees. BMC plant biology. 2021;21(1):1-13.
- Salehi M, Farhadi S, Moieni A, Safaie N, Hesami M. A hybrid model based on general regression neural network and fruit fly optimization algorithm for forecasting and optimizing paclitaxel biosynthesis in Corylus avellana cell culture. Plant Methods. 2021;17(1):1-13.
- Farhadi S, Salehi M, Moieni A, Safaie N, Sabet MS. Modeling of paclitaxel biosynthesis elicitation in Corylus avellana cell culture using adaptive neuro-fuzzy inference system-genetic algorithm (ANFIS-GA) and multiple regression methods. PloS one. 2020;15(8):e0237478.
- Sanchez-Muñoz R, Bonfill M, Cusidó RM, Palazón J, Moyano E. Advances in the regulation of in vitro paclitaxel production: Methylation of a Y-Patch promoter region alters BAPT gene expression in Taxus cell cultures. Plant and Cell Physiology. 2018;59(11):2255-67.
- Sanchez-Muñoz R, Perez-Mata E, Almagro L, Cusido RM, Bonfill M, Palazon J, et al. A novel hydroxylation step in the taxane biosynthetic pathway: a new approach to paclitaxel production by synthetic biology. Frontiers in Bioengineering and Biotechnology. 2020;8:410.
- Kuang X, Sun S, Wei J, Li Y, Sun C. Iso-Seq analysis of the Taxus cuspidata transcriptome reveals the complexity of Taxol biosynthesis. BMC plant biology. 2019;19(1):1-16.
- Kanda Y, Nakamura H, Umemiya S, Puthukanoori RK, Murthy Appala VR, Gaddamanugu GK, et al. Two-Phase Synthesis of Taxol. Journal of the American Chemical Society. 2020;142(23):10526-33. doi: 10.1021/jacs.0c03592.
- Wang T, Chen Y, Zhuang W, Zhang F, Shu X, Wang Z, et al. Transcriptome sequencing reveals regulatory mechanisms of taxol synthesis in Taxus wallichiana var. Mairei. International journal of genomics. 2019.
- He C-T, Li Z-L, Zhou Q, Shen C, Huang Y-Y, Mubeen S, et al. Transcriptome profiling reveals specific patterns of paclitaxel synthesis in a new Taxus yunnanensis cultivar. Plant Physiology and Biochemistry. 2018;122:10-8.
- Zhou X, Zhu H, Liu L, Lin J, Tang K. A review: recent advances and future prospects of taxol-producing endophytic fungi. Applied Microbiology and Biotechnology. 2010;86(6):1707-17. doi: 10.1007/s00253-010-2546-y.
- Walls LE, Malcı K, Nowrouzi B, Li RA, d'Espaux L, Wong J, et al. Optimizing the biosynthesis of oxygenated and acetylated Taxol precursors in Saccharomyces cerevisiae using advanced bioprocessing strategies. Biotechnology and Bioengineering. 2021;118(1):279-93.
- Walker K, Ketchum RE, Hezari M, Gatfield D, Goleniowski M, Barthol A, et al. Partial Purification and Characterization of Acetyl Coenzyme A: Taxa-4 (20), 11 (12)-dien-5α-olO-Acetyl Transferase That Catalyzes the First Acylation Step of Taxol Biosynthesis. Archives of biochemistry and biophysics. 1999;364(2):273-9.

**The specific comments on the pdf file attached**

**Abstract**

**Comment No.1**: please clarify that T5AT is encoded by TDAT gene.

**Response:** Thank you for making the suggestion. The phrase “, encoded by *TDAT*,” was added to the text.

**Comment No.2**: The abstract should be improved with a nice conclusion.

**Response:** The author tried to create a more appropriate conclusion for the abstract.

**Introduction:**

**Comment No.3**: In general introduction is good but the novelty and the aim of study are poorly written. Therefore, introduction should be improved with a better conclusion.

Also, the current studies on TAT characterization or production could be mentioned here.

**Response:** Thanks to the reviewer for the comment. The introduction now includes recent studies on TDAT and taxol that are relevant to the issue. A better conclusion has also been added to the introduction. For this aim the sentences “These results point to TDAT's significance in the PC biosynthetic pathway, making it a promising candidate for PC engineering in *C. avellana* L. In this article, we present novel information on the isolation and characterization of TDAT from methyl jasmonate (MeJA)-induced hazelnut cell suspension, with the goal of better understanding the PC biosynthetic pathway, which could be the subject of future research.” was added to the introduction.

**Materials and methods:**

**Comment No.4**: should not be "bought from"?

**Response:** The sentence “Culture medium salts and vitamins were provided from Merck” was replaced with “Culture medium salts and vitamins were produced from Merck”

**Comment No.5**: the same

**Response:** The modification is like the previous response.

**Comment No.6**: the link does not work, please provide the correct one

**Response:** The link was replaced with <https://www.idtdna.com/pages/tools/oligoanalyzer>. The other links within the text were checked and updated.

**Comment No.7**: this section needs corrections and should be rewritten

**Response:** The author tried to improve the section and rewrite some sentences to get more clarified.

**Comment No.8**: Please clarify here. Was qPCR used for gene expression detection? RT-PCR stands for reverse transcription PCR.

**Response:** In this study, qRT-PCR was not used. This part was clarified with the sentence “*TDAT* gene expression was investigated using reverse transcription PCR (RT-PCR).

**Comment No.9**: should be reworded

**Response:** The sentence was changed to “These primers were used to amplify overlap fragments in order to approve the ORF specification”

**Comment No.10**: The primer names should be same as the table

**Response:** The name of primers replaced with “forward. 1 and reverse. 1” the same as other places within the text.

**Comment No.11**: Fig S1 should be mentioned here

**Response:** The modifications have been considered.

**Comment No.12**: The figure caption should be reworded

**Response:** The modifications have been considered and “the situation of cDNA full length and nested-PCR primers comprising *TDAT* gene.” was replaced with “The position of full-length cDNA and nested-PCR primers containing TDAT gene”.

**Comment No.13**: The links given should be corrected, none of them is working. Some of them are even not in a correct web-link format.

**Response:** All the links in the text have been checked and corrected.

**Comment No.14**: part a and part b should be merged in a single figure as Fig. 2

**Response:** The modifications have been considered.

**Comment No.15**: if possible, a better image could be used

**Response:** The authors have used their best image. So Fig 2 only was merged into a single image and the resolution was improved in his section.

**Comment No.16**: italic

**Response:** The modifications have been considered.

**Comment No.17**: the frame on this motif should be replaced or the letters can be differently colored to emphasize

**Response:** According to the reviewer's comment, the frame was replaced with the ▼ symbol above the motif letters, in this figure. A few errors in the typography of motif HXXXD were also corrected inside the text.

**Comment No.18**: lowercase

**Response:** The modifications have been considered

**Comment No.19**: Please correct the software name which should be PredictNLS

**Response:** Thanks to the reviewer for the comment. The name of the software was correct with PredictNLS.

**Comment No.20**: make sure this figure is legible

**Response:** The author attempted to replace the figure with a more legible one.

**Comment No.21**: part a and b should be merged into a single figure

**Response:** Thanks to the reviewer for the comment. Two separate figures, which are the outputs of the software, were designed into one figure. According to similar references (e.g., Safavi et al., 2019; Elengoe et al., 2014; pandey et al., 2014), parts A and B do not merge into a single image. So, two parts are labeled A and B in this figure, but if the respected reviewer suggests it, the author will merge the two parts in the next revision.

- Safavi, A., Kefayat, A., Sotoodehnejadnematalahi, F. et al. In Silico Analysis of Synaptonemal Complex Protein 1 (SYCP1) and Acrosin Binding Protein (ACRBP) Antigens to Design Novel Multiepitope Peptide Cancer Vaccine Against Breast Cancer. Int J Pept Res Ther 25, 1343–1359 (2019). <https://doi.org/10.1007/s10989-018-9780-z>

-Elengoe, A.; Naser, M.A.; Hamdan, S. Modeling and Docking Studies on Novel Mutants (K71L and T204V) of the ATPase Domain of Human Heat Shock 70 kDa Protein 1. Int. J. Mol. Sci. 2014, 15, 6797-6814. https://doi.org/10.3390/ijms15046797

-Pandey, Saurabh & Negi, Yogesh & Reddy, Subramanyam & S, Krishna Murthy & Arora, Sandeep & Kaul, Dr. Tanushri. (2014). Modeling and phylogenetic analysis of cytosolic ascorbate peroxidase (OsAPX1) from rice reveal signature motifs that may play a role in stress tolerance. Bioinformation. 10. 119-123. 10.6026/97320630010119.

**Discussion:**

**Comment No.22**: discussion should be improved. the most parts look like a repetition of the results section. the importance and potentials of the findings/results should be interpreted and elaborated. the significance of elucidation of the sequence and the structure of this enzyme should be clearly indicated. the recent studies about this enzyme (characterization or heterologous expression in microbial hosts) should be mentioned here and the potential of this study should be discussed.

please see below for other notes.

**Response:** Thanks to the reviewer for the suggestion. The author has been tried to improve the discussion part by adding some new complementary references and rewriting some sentences and also, adding some new sentences to make more explanations and clarifications.

**Comment No.23**: in contrast,

**Response:** The modification “In contrast” has been done.

**Comment No.24**: should be reworded or removed

**Response:** the sentence “this process was the biological process in the pc biosynthesis” was removed.

**Comment No.25**: a single aa cannot be an active site. rather, it should be in active site

**Response:** Thanks for the reviewer comment. Based on the suggestion, the authors changed the sentences and, as a result, the subsequent related sentences. the modified sentences: “Therefore, His-164 is found in the active site of the T5AT protein (C. avellana). The active sites in the T5AT in T. cuspidate were also included His-164 and Asp-373. As a result, it is most likely that Asp-381 is another amino acid in the active site involved in the T5AT (C. avellana).”

**Comment No.26**: activity site is not a correct term. should be active site. this term should be corrected in all parts of the text

**Response:** The “activity site” has been replaced with the “active site” in the whole text.

**Comment No.27**: this part should be improved too

**Response:** The modification was made according to the suggestion.

**Comment No.28**: conclusion is poorly written, should be improved

**Response:** The author tried to improve the conclusion by replacing and rewriting some sentences.

**Comment No.29**: not a clear statement, should be improved/clarified

**Response:** The sentence “It can be deduced that the taxadien-5α-ol-O-acetyltransferase enzyme has undergone many changes during development.” was replaced with “It can be assumed that the taxadien-5α-ol-*O*-acetyltransferase enzyme has undergone numerous changes during its evolution”.

**Comment No.30**: This gene was in the primary PC biosynthetic pathway.

**Response:** the mentioned sentence was removed.

**Comment No.27**: Please correct the underlined parts in fig 1

**Response:** Fig 1 was modified according to the suggestion.

**Response to Comments by Reviewer# 2**

We would like to thank the Reviewer for his/her constructive comments and suggestions. Based on his/her remarks, various revisions have been made to improve the quality of the manuscript. Our responses to the review comments are presented next.

**Abstract**

**Comment No.1**: Line no. 16 – 18: ‘and tissue cultures of Taxus 16 species and’ not correlating here. Reframe the sentence.

**Response:** Thanks for the reviewer comment. According to the suggestion, the authors improved the sentence with “Natural materials including tissue cultures of *Taxus* species and, most recently, hazelnut (*Corylus avellana* L.) represent the most promising sources of this compound.”

**Comment No.2**. Line no. 20: change as ‘this study was undertaken to …..

**Response:** The sentence “this study was undertaken to…” has been replaced with the “this paper aimed to identify *taxadiene-5α-ol-O-acetyltransferase* (*TDAT*) in hazelnut.”

**Introduction**

**Comment No.3**: Line no. 44 – 46: Sentence starting as ‘In particular……’ rewrite the sentence

**Response:** the mentioned sentence was improved as follows: “Particularly, hazelnut (*Corylus avellana* L.) [6], some microorganisms like yew endophytic fungi,{Wani, 1971 #44;Vasundhara, 2019 #73} and hazelnut endophytic fungi [7, 8] are the other raw resources of the PC production.”

**Comment No.4**: Line no. 55 – 58: Sentence starting as ‘The deep and detailed understanding’ rewrite the sentence.

**Response:** The mentioned sentence was replaced with “A comprehensive understanding of the PC biosynthetic pathway in hazelnut, particularly the genes encoding rate-limiting enzymes and the enzymes catalyzing these reactions, are influential factors for developments in metabolic engineering and and for the production of PC to be significantly increased”

**Comment No.5**: Line no. 65: Change as ‘The initial main precursor of PC’

**Response:** The sentence was modified according to the comment.

**Materials and methods**

**Comment 6**: Line no. 88: ‘were procured from Merck’

**Response:** The modification was considered

**Comment 7**: Line no. 89: ‘were procured from Merck’

**Response:** The modification was considered

**Comment 8**: Line no 94: dissolving

**Response:** The modification was considered

**Results**

**Comment 9**: Line no. 195: (non-elicited)

**Response:** The modification was considered

**Comment 10**: Line no. 196 and 197: Remove (0)

**Response:** The modification was considered

**Comment 11**: Line no. 200: change as ‘for 1423 bp and were subjected to sequencing.

**Response:** The modification was considered

**Comment 12**: Line mo. 305 – 308: consider rewriting the sentence

**Response:** The sentence was modified as follows: “Primarily, similar and most close sequences to the T5AT sequence were obtained using the protein BLAST database. The hydroxycinnamoyl-COA shikimate/quinate hydroxycinnamoyl transferase enzyme, which it’s 3D-structure (PDB code; 4G0B) was identified by Lallemand et al. [44], was considered as a template for the T5AT protein.”

**General comment:**

**Comment 13**: The authors have to improve the language in the entire manuscript to meet the journal standard.

**Response:** Thank you very much to the reviewer. The author attempted to proofread and refine the entire manuscript's grammar to meet the journal’s standard. The grammatical revisions have been marked with track changes inside the text.

Best Regards

6- PLOS authors have the option to publish the peer review history of their article (what does this mean?). If published, this will include your full peer review and any attached files.

If you choose “no”, your identity will remain anonymous but your review may still be made public.

**Response:** No.
