# Supplementary material for: Identification and in-silico characterization of taxadien-5α-ol-O-acetyltransferase (TDAT) gene in Corylus avellana L
Source: PLoS One. 2021 Aug 27;16(8):e0256704. doi: 10.1371/journal.pone.0256704 (PMC8396717; doi:10.1371/journal.pone.0256704)
Supplement: Attachment — Submitted filename: responses to reviewer.docx [file pone.0256704.s013.docx]

Dear Editor-in-Chief,

We would like to deeply thank the editorial office and the reviewers for the constructive comments which helped us to improve the paper and thank you for giving us the opportunity to submit a revised draft of our manuscript titled "Identification and *in-silico* Characterization of *taxadien-5α-ol-O-acetyltransferase (TDAT*) Gene in *Corylus avellana* L". The authors tried to address all reviewers’ comments in the revised manuscript. We hope our revision has improved the paper to a level of their satisfaction. The revisions in the manuscript have been marked highlighted with track changes in a file labeled ‘Revised manuscript with track changes.

Enclosed, please find the revised version of the manuscript entitled:

“Identification and *in-silico* Characterization of *taxadien-5α-ol-O-acetyltransferase (TDAT*) Gene in *Corylus avellana* L.”

By:

Mona Raeispour Shirazi, Sara Alsadat Rahpeyma, Sajad Rashidi Monfared, Jafar Zolala and Azadeh Lohrasbi-Nejad^1^

Sincerely yours,

Dr. Sara Alsadat Rahpeyma

Assistant Professor of Plant Breeding

Department of Agricultural Biotechnology

Faculty of Agriculture

Shahid Bahonar University of Kerman

Iran

Response to Comments by Reviewer 3

We'd like to express our gratitude to the Reviewer for his or her constructive comments and suggestions. Based on his/her remarks, various revisions were made to improve the quality of the manuscript. The modifications were highlighted with track changes through the revised manuscript. The next sections contain our responses to the dear reviewer comments.

**Comment No.1**: The authors shall rephrase all the RT-PCR into sqRT-PCR, which misinterpret that the authors had performed real-time quantitative PCR.

**Response:** Much appreciated to the reviewer for the kind comments on the manuscript. In this study, the novel EST assembly strategy has been applied for identification of *TDAT* gene, and subsequently reverse transcription (RT)-PCR technique was developed to isolate *TDAT* gene from cDNA. As you know, the term “RT-PCR” is used for amplification, and semi-quantitative reverse transcription (sqRT-PCR) is a simple and specific method for relative quantitative RNA and amplification and determination of the quantity of PCR products. Since in this paper, the quantity measure was not intended, so it seems that RT-PCR is a more reasonable term in this case. It’s also worth to noting that the term “RT-PCR” was use in different studies *(e.g.* ***-*** *Molecular cloning and expression analysis of glutathione reductase gene in Chlamydomonas sp. ICE-L from Antarctica, Marine Genomics, Volume 5, 2012. - Dongmei Yin, Dian Ni, Lili Song, Zhiguo Zhang, Isolation of an alcohol dehydrogenase cDNA from and characterization of its expression in chrysanthemum under waterlogging, Plant Science, Volume 212, 2013.* ***-*** *Chun-Hong Li, Yong-Qing Zhu, Yu-Ling Meng, Jia-Wei Wang, Ke-Xiang Xu, Tian-Zhen Zhang, Xiao-Ya Chen, Isolation of genes preferentially expressed in cotton fibers by cDNA filter arrays and RT-PCR, Plant Science, Volume 163, Issue 6, 2002.* ***-*** *Iwao, Kyoko, Watanabe, Takashi, Fujiwara, Yoshiyuki, Takami, Koji, Kodama, Ken, Higashiyama, Masahiko, Yokouchi, Hideki, Ozaki, Kouichi, Monden, Morito, Tanigami, Akira. Isolation of a novel human lung-specific gene, LUNX, a potential molecular marker for detection of micrometastasis in non-small-cell lung cancer. International Journal of Cancer. Int. J. Cancer)*

**Comment No.2**: Based on figure 2, all lanes 2, 3 and 4 showed the smear, and, intriguingly, only the 150 uM resulted in the amplification of the TDAT gene.

**Response:** As you know, the smear is commonly seen in the background of agarose gel electrophoresis of RT-PCR products. Moreover, In the case of this study, it was very difficult to obtain RNA samples with high concentrations of total RNA from cultured cells of hazelnut. For this reason, we had to use maximum amounts of total RNA samples and RT products in RT and subsequent PCR reactions. These conditions were the main reasons for the observation of smear in the background of agarose gel. The authors also, carried out this experiment aimed at significant expressing of the TDAT gene, several times and it was confirmed from the results that the expression was not detected at low concentrations of elicitors. About the amplification of desired PCR products only in reactions with RNA from 150 µM MeJA, Nevertheless, according to the dear reviewer comment, and to avoid of the claims related with comparative experiments, the phrase ‘while TDAT expression was induced at only 150 µM of MeJA’ was revised with the phrase ‘and it was perceivable at higher concentration of MeJA’(P8 line219 and 220), and the phrase “of an exact concentration” was deleted from P19-line 507

**Comment No.3**: What is the dark line present in figure 2 between lane 3 and lane 4? The authors claimed that gel cropped from different gels was differentiated by white lines; however, no such differentiation exists in this figure.

**Response:** That's correct, and thanks for bringing it out. The trimmed areas of figure 2 had been distinguished in the original figure (S1_ row_ images.pdf) attached to the supplementary information. So the change was considered, and the dark line was replaced with white lines. During the previous edition, the figure 2 was also modified in response to feedback from reviewers.

**Comment No.4**: The authors claimed that they had purified the PCR product obtained from lane 5. Was it gel elution or purification?.

**Response:** It was gel purification using Ron’s Gel Extraction Kit (BIGRON, cat no.: 802501) for 1423 bp. It is mentioned at page 7 line 155.

**Comment No.5**: P7 L155-authors mentioned as gel extraction- these details may be precisely given in the results section..

**Response:** Many thanks for the comment. The phrase “using Ron’s Gel Extraction Kit (BIGRON, cat no.: 802501)” was deleted from the results.

**Comment No.6:** Moreover, lane 7 does not seem to contain the purified products, instead, it looks more smear than the unpurified products (Lane 5). The authors explain this context.

**Response:** Many thanks for these precise comments. As previously stated, the 1423 bp amplified DNA fragment was purified from an agarose gel using Ron’s Gel Extraction Kit (BIGRON, cat no.: 802501). Despite all the optimizations, the target PCR fragment was so thin that we had to use a large gel slice in the purification process. The smear in the background of Lane 7 is caused by utilizing the maximum amount of an agarose gel in the purification process. It's also worth noting that we designed PCR primers using the TDAT assembly sequence derived from an EST library as a template. To amplify the complete CDS in PCR reactions, we had to locate the primers at the end of the target sequence. As a result, we faced significant challenges in detecting and overcoming false priming and other qualitative criteria in designed primers. Undesirable bands have been amplified in PCR reaction possibly due to the false priming of PCR primers. However, we obtained a clean PCR product when using the purified PCR product as the template in nested-PCR reactions (S3 Fig, page 9).

**Comment No.7**: Based on figure 2, the presence of TDAT amplicon in the lanes 2, 3 an 4 can not be ruled out. There might be some problem with the purification of PCR amplicons from the gel. Collectively, to avoid all these misperceptions, the authors are highly recommended to perform real-time quantitative PCR analysis to detect the relative mRNA level of TDAT gene at 0, 50, 100 and 150 uM of MeJa.

**Response:** Thank you so much for your detailed comment. Certainly, qRT-PCR is most reliable choice for comparative expression studies of TDAT gene. However, as stated in the title, the major goal of this study was identification and sequence analysis of the target gene in hazelnut. Therefore, we focused our efforts on providing a detectable source of TDAT mRNA for use in gene isolation assays. We performed many PCR reactions on different amounts of RT samples from different cell samples. Every time, we found the target mRNA only in cells induced with 150 µM MeJA. With this reason, the work is only based on common RT-PCR reactions not semi quantitative or quantitative ones. It was enough to our purpose of the study. We checked throughout the manuscript and made essential revisions to ensure there are no claims related with comparative experiments. Please refer to P8 line 219 and 220; P19-line 507 in the revised manuscript.

**Comment No.8**: The number of figures may be reduced. Few figures may be shifted to the Supplementary file. For instance, Figs 1, 2, 3, 5, 7

**Response:** The modification was considered and the fig 1, 2, 5 and 7 were shifted to the supplementary file.

**Comment No.9**: P4 L96: What is the content/quantity of callus taken to generate suspension culture as per the given protocol, which may be provided in the methods section.

**Response:** Almost 1 g white and friable callus was taken and suspended in 30 mL liquid media to generate suspension culture. The modification was added to the text.

**Comment No.10**: Which term the authors imply: produced or procured?

**Response:** The term produced was implied. It was recommended with the other reviewer.

**Comment No.11**: µm

**Response:** Thanks to the reviewer for the comment. The modifications have been considered.

**Comment No.12**: Primer sequences may be given in the table

**Response:** The primers’ sequence was added in S1 table.

**Comment No.13**: P19 L454-455: This claim warrants the use of qPCR analysis, considering the inconsistent result of the given sqRT-PCR gel pictures.

**Response:** Thanks for these precise comment from the reviewer. The authors did not do quantitative analysis because their efforts were focused on obtaining a reliable and detectable supply of TDAT mRNA for use in gene isolation assays, as stated in earlier comments. Using different concentrations of elicitors, in this paper, indicates valuable results of using elicitation for identification of low-expressed taxol genes in hazelnut or even other genes. Please consider our more explanations under the reviewer suggestions about qPCR.
